# Leader peptide removal in lasso peptide biosynthesis based on penultimate isoleucine residue

**DOI:** 10.3389/fmicb.2023.1181125

**Published:** 2023-07-10

**Authors:** Yuwei Duan, Weijing Niu, Linlin Pang, Da-Shuai Mu, Zong-Jun Du, Youming Zhang, Xiaoying Bian, Guannan Zhong

**Affiliations:** ^1^Helmholtz International Laboratory for Anti-Infectives, State Key Laboratory of Microbial Technology, Shandong University, Qingdao, China; ^2^Marine College, Shandong University, Weihai, China; ^3^CAS Key Laboratory of Quantitative Engineering Biology, Shenzhen Institute of Synthetic Biology and Faculty of Synthetic Biology, Shenzhen Institute of Advanced Technology, Chinese Academy of Sciences, Shenzhen, China; ^4^Suzhou Research Institute of Shandong University, Suzhou, China

**Keywords:** lasso peptide, biosynthesis, post-translational modification, phosphorylation, peptidase, penultimate isoleucine residue

## Abstract

Lasso peptides are ribosomally synthesized peptides that undergo post-translational modifications including leader peptide removal by B (or the segregated B1 and B2) proteins and core peptide macrolactamization by C proteins to form a unique lariat topology. A conserved threonine residue at the penultimate position of leader peptide is hitherto found in lasso peptide precursors and shown to be a critical recognition element for effective enzymatic processing. We identified a lasso peptide biosynthetic gene cluster (*bsf*) from *Bradymonas sediminis* FA350, a Gram-negative and facultatively prey-dependent bacterium that belongs to a novel bacterial order *Bradymonadales* in the class *Deltaproteobacteria*. The kinase BsfK specifically catalyzes the phosphorylation of the precursor peptide BsfA on the Ser3 residue. BsfB1 performs dual functions to accelerate the post-translational phosphorylation and assist BsfB2 in leader peptide removal. Most importantly, the penultimate residue of leader peptide is an isoleucine rather than the conserved threonine and this isoleucine has a marked impact on the phosphorylation of Ser3 as well as leader peptide removal, implying that BsfB1 and BsfB2 exhibit a new substrate selectivity for leader peptide binding and excision. This is the first experimentally validated penultimate isoleucine residue in a lasso peptide precursor to our knowledge. *In silico* analysis reveals that the leader peptide Ile/Val(-2) residue is rare but not uncommon in phosphorylated lasso peptides, as this residue is also discovered in *Acidobacteriaceae* and *Sphingomonadales* in addition to *Bradymonadales*.

## Introduction

Lasso peptides are a group of ribosomally synthesized and post-translationally modified peptides (RiPPs) with a characteristic lariat topology, in which the *C*-terminal tail threads through the macrolactam ring formed by the *N*-terminal amino group and the carboxylic acid side chain of an aspartate or a glutamate located at position 7–9 of the core peptide via an isopeptide bond (Hegemann et al., 2015; Martin-Gómez and Tulla-Puche, 2018). The number and position of disulfide linkages are used to categorize lasso peptides into four classes, among which class II is the most frequently encountered and features no disulfide bridge, and the lasso topology is stabilized only by steric interactions above and below the ring (Zhao et al., 2016; Martin-Gómez and Tulla-Puche, 2018; Cheng and Hua, 2020; Hegemann et al., 2020; Wang et al., 2021). Generally speaking, the biosynthesis of lasso peptides requires at least three proteins, including a precursor peptide (A), a cysteine protease (B) with homology to transglutaminases, and an adenosine triphosphate (ATP)-dependent macrolactam synthetase (C) homologous to asparagine synthetases (Supplementary Figure S1). Many lasso peptide biosynthetic gene clusters (BGCs) also contain a fourth gene encoding an ABC-transporter (*D*), which is responsible for excretion and self-resistance. *B* genes from actinobacteria and firmicutes are usually split into two genes: *B1* encodes the *N*-terminal RiPP precursor recognition element (RRE) domain with homology to PqqD family proteins, while *B2* encodes the *C*-terminal protease domain that cleaves the leader peptide of the precursor, after which the remanent core peptide is macrolactamized to form the mature lasso peptide (Burkhart et al., 2015; Hegemann et al., 2015; Martin-Gómez and Tulla-Puche, 2018).

Beyond the class-defining leader peptide removal and core peptide macrolactamization, nine unique post-translational modifications (PTMs) are reported in total in the biosynthesis of lasso peptides up to now (Duan et al., 2022), among which the (poly)phosphorylation, methylation, hydroxylation, and epimerization are verified to act on the linear precursor peptide ahead of leader peptide removal (Zhu et al., 2016b,c; Su et al., 2019; Feng et al., 2020; Zhang and Seyedsayamdost, 2020). Besides, the B1 proteins were proven to enhance the turnover rates for epimerization and hydroxylation (Feng et al., 2020; Zhang and Seyedsayamdost, 2020), hinting at the dual functions for RREs.

**Figure 1:**
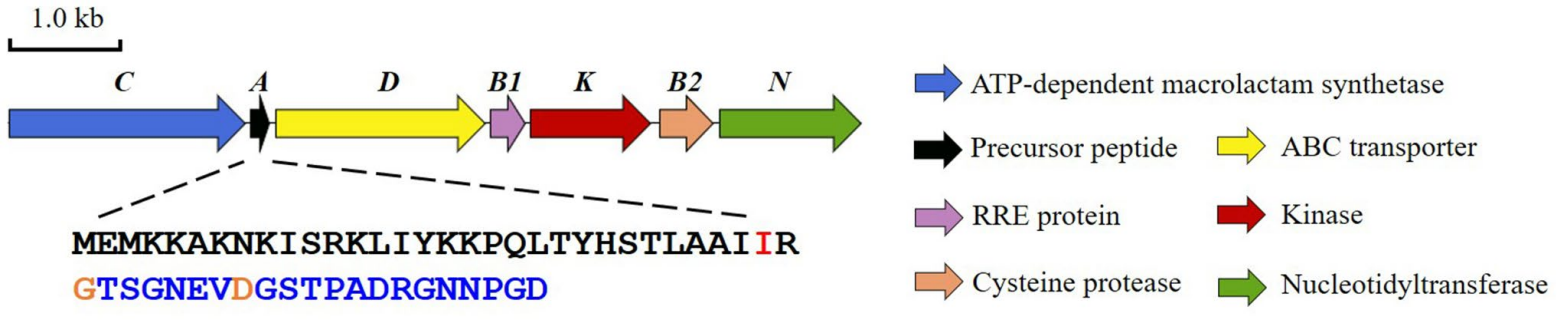
The lasso peptide BGC (*bsf*) from *Bradymonas sediminis* FA350.

*Bradymonas sediminis* FA350 is a Gram-negative, rod-shaped, and facultatively prey-dependent bacterium isolated from coastal sediments in Weihai, China. Phylogenetic analysis based on 16S rRNA gene sequences showed that this strain belongs to a novel bacterial order *Bradymonadales* and a novel family *Bradymonadaceae* in the class *Deltaproteobacteria* (Wang et al., 2015; Mu et al., 2020). Sequencing of the genome sequence of *B. sediminis* FA350 (accession No. NZ_CP030032.1) and subsequent search for natural products BGCs allowed the identification of a lasso peptide BGC, which we subsequently named *bsf* (Figure 1; Supplementary Figure S2; Wang et al., 2019). The candidate cluster consists of 7 open reading frames (ORFs) spanning 7.2 kb, bordered at one end by an FG-GAP repeat protein and at the other by ribosomal proteins, presumably not involved in lasso peptide biosynthesis. Apart from the conserved genes encoding the precursor peptide (*bsfA*), a discrete RRE (*bsfB1*), a leader peptidase (*bsfB2*), a lasso peptide cyclase (*bsfC*) and an ABC-transporter (*bsfD*), two additional genes encoding a putative kinase (*bsfK*) and a predicted nucleotidyltransferase (*bsfN*) have been found in this BGC as well. Herein, we characterized the activities of BsfK, BsfB1, and BsfB2.

## Materials and methods

### Materials, bacterial strains, and plasmids

Biochemicals and media were purchased from Sinopharm Chemical Reagent Co. Ltd. (China) or Oxoid Ltd. (United Kingdom) unless stated otherwise. Enzymes were purchased from New England Biolabs Ltd. (United Kingdom) except *ApexHF* HS DNA polymerase for high-fidelity amplification from Accurate Biology Co. Ltd. (China). Chemical compounds and reagents were purchased from Bide Pharmatech Ltd. (China), Macklin Biochemical Technology Co., Ltd. (China) and J&K Scientific Ltd. (China) unless stated otherwise. Gene synthesis and codon optimization were performed at GENEWIZ, Inc. (China). Primer synthesis and DNA sequencing were performed at Shanghai Sangon Biotech Co. Ltd. (China). Primers used in this study are summarized in Supplementary Figure S6.

### Analyses

HPLC analysis was carried out on a Thermo Fisher Dionex UltiMate 3,000 UHPLC system (Thermo Fisher Scientific Inc., United States) using an Acclaim TM RSLC 120 C18 column (2.1 × 100 mm, 2.2 μm, 120 Å, Thermo Fisher Scientific Inc., United States) by gradient elution of solvent A (H_2_O containing 0.1% formic acid) and solvent B (CH_3_CN containing 0.1% formic acid) with a flow rate of 0.3 mL/min over a 25 min period as follows: T = 0 min, 5% B; T = 3 min, 5% B; T = 18 min, 95% B; T = 22 min, 95% B; T = 23 min, 5% B; and T = 25 min, 5% B (mAU at 220 nm). ESI-MS was performed on a Bruker AmaZon SL Ion Trap LC/MS spectrometer (Bruker Co. Ltd., Germany), and the data were analyzed using Bruker Daltonics DataAnalysis. ESI-HRMS analysis was carried out on a Bruker High Resolution Q-TOF mass spectrometry (impactHD; Bruker Co. Ltd., Germany) and the data were analyzed using Bruker Daltonics DataAnalysis. Tandem MS analysis was performed by collision-induced dissociation (CID) using He as collision gas.

### Sequence analysis

ORFs were identified using the FramePlot 4.0beta program.^1^ The deduced proteins were compared with other known proteins in the databases using available BLAST methods.^2^ Amino acid sequence alignments were performed using the Strap program.^3^ Conserved motif identification was performed by WebLogo 3.^4^ Phylogenetic analysis was conducted by Mega 11 software.

### General nomenclature

For peptide residue numbering, the positive numbers indicate amino acid residues in the core peptide counting forward from the leader peptide cleavage site. Negative numbers indicate residues in the leader peptide counting backward from the leader peptide cleavage site.

### Cloning and heterologous expression of *bsf* cluster

The *bsf* cluster was directly cloned from the genomic DNA of *B. sediminis* FA350 via Red/ET recombineering in *Escherichia coli*. The direct cloning experiments were performed according to the previous protocols (Fu et al., 2012; Wang et al., 2016, 2018). Briefly, genomic DNA was digested by appropriate restriction enzyme (*Bgl*II) and purified by ethanol precipitation. The digested genomic DNA, p15A vector with homologous arms, T4 polymerase, and buffer were mixed and reacted in a thermocycler with the following program: 25°C, 60 min; 75°C, 20 min; 50°C, 60 min. The mixture was then electroporated into the l-arabinose induced *E. coli* GB05-dir competent cells and plated on appropriate Luria-Bertani (LB)-agar plates to incubate at 37°C overnight. The *bsf* region (approximate 22.67 kb) was cloned into the corresponding p15A-cm vectors in *E. coli*, and several constitutive promotors such as P_Tn5-Km_, P_apra_, or the inducible promoter P_tet_ were subsequently inserted upstream to *bsfC*, respectively, leading to the constructions of p15A-cm-Tn5-km-bsf, p15A-cm-P_apra_-bsf, and p15A-cm-P_tet_-km-bsf. Then, the plasmids were electroporated into the heterologous hosts *E. coli* BL21(DE3) or *E. coli* GB2005. In order to heterologous express the BGC in *Burkholderiales* hosts, the chloramphenicol resistance gene was then replaced with phiC31-site-specific integrase by recombineering, resulting the construction of p15A-phiC31-amp-P_apra_-bsf. This plasmid was then electroporated into *Schlegelella brevitalea* DSM 7029 or introduced into the specific chromosomal site of *Burkholderia gladioli* ATCC10248 and *Burkholderia thailandensis* E264 with the help of donor strain *E. coli* WM3064, which is auxotrophic for diaminopimelic acid (Bonis and Gralnick, 2015).

The obtained colonies were verified by sequencing. The correct transformants containing *bsf* cluster were cultured overnight and inoculated into 50 mL antibiotic supplemented LB, M9, or CYMG medium in a 250 mL flask and incubated at 30°C, 200 rpm for 4 days. The resin Amberlite XAD-16 was added into the culture and further incubated for another day. The cells and resins were collected by centrifugation and extracted with 40 mL MeOH for 2 h. The supernatant was concentrated *in vacuo* and the resulting residue was dissolved in 1 mL MeOH for further metabolic analysis by HPLC-MS or HPLC-HRMS.

### Gene sequences of the codon optimized *bsfA, bsfB1, bsfB2,* and *bsfK*

*bsfA* (accession No. WP_162687377.1), inserted into the *BamH*I-*Hind*III site of the plasmid pRSFDuet-1ATGGAAATGAAAAAAGCTAAAAACAAAATCTCTCGTAAACTGATCTACAAAAAACCGCAGCTGACCTACCACTCTACCCTGGCTGCTATCATCCGTGGTACCTCTGGTAACGAAGTTGACGGTTCTACCCCGGCTGACCGTGGTAACAACCCGGGTGACTAA

*bsfB1* (accession No. WP_162687378.1), inserted into the *Nde*I-*Xho*I site of the plasmid pET28aATGCGTGAAACCCCGATGACCGAAAACCTGTTCCCGCGTGACGCTGTTTTCTCTATCCGTGAAGACCTGGTTGTTGAACAGGTTGACGACGAATTCCTGGTTCTGGACCTGCGTGGTAACGAATACTTCGGTCTGAACGCTGTTGCTCGTCACATCTGGGCTGCTATCGACGCTGGTGACTCTCTGGCTGCTATCGCTGACTCTGTTTGCGAACGTTTCGAAGTTGAACGTGAACGTGCTGCTACCGACGTTGCTGACTTCATCGCTAACCTGCTGGAACAGCGTCTGGTTTCTCGTGTTGACGCTTAA

*bsfB2* (accession No. WP_111331306.1), inserted into the *Nde*I-*Xho*I site of the plasmid pET28aATGAAACCGATCCACCCGATCGCTCAGGCTGAAATGCTGGTTGAAGTTGCTATCGCTCGTGTTCTGTCTGAATTCCTGTCTATCGAAGACCTGCTGCGTCTGGTTGGTGAAGCTGCTCGTATCGCTCAGCAGTGGCCGGCTCGTTCTTGCGTTGCTTCTCCGTCTGCTGAAGCTATCAACGAAACCGCTGCTCTGTCTGAACTGCTGGCTCACCGTGCTTCTCGTCTGGTTCCGGGTGCTCAGTGCCTGCAGCGTGCTCTGGCTGGTCGTGTTTGGCTGGCTCGTCGTGGTATCGCTTCTGAAATCGTTGTTGGTTTCCGTAAACGTGGTGTTCTGGAAGGTCACGCTTGGCTGGAAGTTATGTCTCCGGACGGTCTGATCGAACTGTTCAACAACGCTGACGACGGTTACCGTGAATCTTTCCGTGAAGTTGCTGCTTAA

*bsfK* (accession No. WP_204355063.1), inserted into the *Nde*I-*Xho*I site of the plasmid pET28aATGGAAAACACCGACCGTCTGGTTCGTTACCGTGCTTTCGGTCGTGACATCGACGGTCCGGCTGGTCTGTCTCTGTCTCCGGCTCCGGCTGAAGCTGCTCCGGGTGAAGTTCCGGTTCTGCGTCTGCAGCCGGACGCTTCTCTGCGTGTTTTCATCGACGAAAACCTGCCGCCGCTGCTGCACAACGTTGAAGACTACCCGGGTGGTCCGAAATTCTACGTTTGGCAGGAAGGTGAAGCTGTTGGTGTTCAGTACGACCGTTGGCGTACCCGTCTGATCCCGGGTGAAGGTCGTATCGACTTCGCTGAACTGCCGCCGTCTGGTCCGAAACGTGTTGAAGACGCTGGTGACGAATACGGTCGTTTCCGTTTCTCTCTGGCTATGGAACGTGTTTTCCTGCCGCTGTACGCTCTGTTCTCTATGCCGGACGCTGTTGCTCTGCACGGTTCTGCTGTTGTTCTGAACGGTGAAGCTTTCCTGTTCATCGGTCGTTCTGGTGCTGGTAAATCTACCACCGCTTACGAATTCGTTCGTCGTGGTGCTACCCTGCTGGCTGACGACCTGATCGTTGCTGACGTTGCTCGTGGTATCGCTCTGGGTGGTGCTCCGACCCTGCGTCTGTGGAAAGGTGAAGGTGCTCTGCCGGAAGCTCAGGAAGACCGTTCTCTGTGGCGTCACGACGCTTCTAAACGTTGGTTCCGTATCCCGGCTGAACGTGGTGCTGCTTCTGCTGTTCCGATCGCTGCTATCGTTATGCTGGACCCGGACACCCTGGGTGGTCAGCGTGACGTTCTGCCGGGTCTGGAAACCTCTCCGCAGCGTAAAGCTCTGACCGACCTGCTGGGTCAGACCTTCGACCTGTCTCACGGTACCCCGGAATGGATGGTTGCTCGTTTCCGTAACACCGCTCGTCTGATCCGTGAATACCCGTTCTACCACTTCCGTTACGTTAAATCTGCTGACGGTAAACCGACCCACATGGACGCTCTGTACCAGGCTATCGTTGGTCTGGCTTCTAAATAA

### Construction of the plasmid pRSFDuet-1 + *bsfA* + *bsfK*

The DNA fragment containing *bsfK* was amplified by PCR with pET28a + *bsfK* as the template. After purification by agarose gel electrophoresis and restrictive digestion, the *bsfK* fragment was inserted into the *Nde*I-*Xho*I site of the plasmid pRSFDuet-1 + *bsfA* to yield the recombinant plasmid pRSFDuet-1 + *bsfA + bsfK*.

### Construction of the plasmid pACYCDuet-1 + *bsfB1* and pCold-TF + *bsfB2*

The DNA fragment containing *bsfB1* or *bsfB2* was amplified by PCR, respectively. The fragments were inserted into the *Nco*I-*Hind*III site of pACYCDuet-1 and the *Nde*I-*Xho*I site of pCold-TF to yield the recombinant plasmids pACYCDuet-1 + *bsfB1* and pCold-TF + *bsfB2*, respectively.

### Expression and purification of BsfA

The plasmid pRSFDuet-1 + *bsfA* was transferred into *E. coli* BL21 (DE3) for expression. BsfA, fused to an *N-*terminal 6 × His tag was expressed at 20°C for 24 h with 100 μM isopropyl-β-d-thiogalactopyranoside (IPTG, added at OD_600_ = 0.6) induction and shaking at 200 rpm. Cells were harvested by centrifugation at 4°C and re-suspended in buffer A containing 6 M guanidine hydrochloride, 20 mM NaH_2_PO_4_ (pH 7.5), 500 mM NaCl, 0.5 mM imidazole. After disruption by a low-temperature, ultra-high-pressure homogenizer, the insoluble material was removed by centrifugation at 20,000 g and 4°C for 1 h. The soluble fraction was subjected to purification using a Ni Bestarose FF column (Bestchrom, China). Briefly, the column was equilibrated with at least 2 column volumes of binding buffer. Then, the pre-treated sample was loaded onto the Ni Bestarose FF column, and washed with elution buffer containing a low concentration of imidazole (25 mM) to remove the impurities. The sample was further eluted with elution buffer containing a high concentration of imidazole (1 M) to obtain the purified BsfA peptide. The eluted fractions were desalted by reversed phase (RP) HPLC on a Shimadzu LC-20AT system (Shimadzu Corporation, Japan) equipped with a ReproSil 300 C4 column (10 × 250 mm, 10 μm, 300 Å, Dr. Maisch, Germany). The solvent used for RP-HPLC separation was deionized water with 0.1% trifluoroacetic acid (TFA; solvent A), and acetonitrile with 0.1% TFA (solvent B). A gradient was applied: 5% B for 15 min, ramp up to 100% B over 50 min, hold at 100% B for 7 min, and ramp to 5% B over 5 min. Eluted fractions were monitored by UV absorbance at 220 nm. Desalted precursor peptides were lyophilized and stored at −20°C. The peptide was validated by HPLC-ESI-(HR)MS analysis on an Acclaim TM RSLC 120 C18 column (2.1 × 100 mm, 2.2 μm, 120 Å, Thermo Fisher Scientific Inc., USA) by gradient elution of solvent A (H_2_O containing 0.1% formic acid) and solvent B (CH_3_CN containing 0.1% formic acid) with a flow rate of 0.3 mL/min over a 25 min period as follows: T = 0 min, 5% B; T = 3 min, 5% B; T = 18 min, 95% B; T = 22 min, 95% B; T = 23 min, 5% B; and T = 25 min, 5% B (mAU at 220 nm).

### Expression and purification of phosphorylated BsfA

The plasmid pRSFDuet-1 + *bsfA + bsfK* was electroporated solely or co-electroporated with pACYCDuet-1 + *bsfB1* (without *N*-terminal 6 × His-tag) into *E. coli* BL21 (DE3) for expression. The precursor peptides were purified, desalted, lyophilized, and validated according to the procedures described above for unmodified BsfA precursor peptide.

### Expression and purification of BsfK

The plasmid pET28a + *bsfK* was transferred into *E. coli* BL21(DE3) and expressed according to the procedures described above for BsfA. Cells were harvested by centrifugation at 4°C and re-suspended in lysis buffer containing 50 mM Tris–HCl (pH 8.0), 300 mM NaCl, 5 mM imidazole and 10% (v/v) glycerol. After disruption by a low-temperature, ultra-high-pressure homogenizer, the insoluble material was removed by centrifugation at 20,000 g and 4°C for 1 h. The soluble fraction was subjected to purification using a Ni Bestarose FF column. The column was equilibrated with at least 2 column volumes of binding buffer. Then, the pre-treated sample was loaded onto the Ni Bestarose FF column, and washed with binding buffer. The sample was further eluted with elution buffer using a stepwise gradient containing different concentrations of imidazole (10–500 mM). The elution fractions were determined by 10% sodium dodecyl sulfate polyacrylamide gel electrophoresis (SDS-PAGE) analysis, and the fractions containing the recombinant protein were desalted using a PD-10 Desalting Column (GE Healthcare, United States) into storage buffer containing 50 mM Tris–HCl (pH 8.0), 100 mM NaCl, 10% (v/v) glycerol, and 1 mM DTT. The resulting protein was concentrated and stored at −80°C. The purity of the protein was determined by SDS-PAGE analysis, and the concentration was determined by the Bradford assay using bovine serum albumin (BSA) as the standard.

### Expression and purification of BsfB1 and TF-BsfB2

The plasmids pET28a + *bsfB1* and pCold-TF + *bsfB2* were transferred into *E. coli* BL21 (DE3) for expression, respectively. The expression temperature was 15°C for TF-BsfB2 instead of 20°C for BsfK and BsfB1. The proteins were purified to homogeneity, and concentrated according to the procedures described above for BsfK.

### Site-directed mutagenesis of *bsfA*

Plasmids containing site-directed mutations were generated by PCR amplification with pRSFDuet-1 + *bsfA* or pRSFDuet-1 + *bsfA + bsfK* as the template. Then, 1 μL of *Dpn*I enzyme was added to the PCR system and incubated at 37°C for 3 h to remove the template. And the PCR system was electroporated into *E. coli* BL21 (DE3). After sequencing to validate the fidelity, the resulting precursor peptide variants were expressed individually, or co-expressed with BsfK and BsfB1 in *E. coli* BL21 (DE3), and purified to homogeneity, desalted, and lyophilized according to the procedures described above for the native precursor peptides.

### Site-directed mutagenesis of *bsfK*

Plasmids containing site-directed mutations were generated by PCR amplification with pET28a + *bsfK* as the template. Then, 1 μL of *Dpn*I enzyme was added to the PCR system and incubated at 37°C for 3 h to remove the template. The PCR system was electroporated into *E. coli* BL21 (DE3). After sequencing to validate the fidelity, the resulting BsfK variants were expressed in *E. coli* BL21 (DE3), purified to homogeneity and desalted according to the procedures described above for the native protein.

### Trypsin digestion assay

The (phosphorylated) precursor peptide BsfA (or the BsfA variants) was dissolved in 50 mM NH_4_HCO_3_ buffer (pH 7.8) to a concentration of 500 μM. 20 μg trypsin (Promega Biotech Co. Ltd., United States) was dissolved in 20 μL resuspension buffer (50 mM acetic acid) and heated at 30°C for 15 min before utilization for maximum activity. 10 μL resuspended trypsin was added to a total volume of 50 μL BsfA reaction system, followed by incubation at 37°C for 2 h. The reaction was quenched by adding an equal volume of CH_3_OH. After removal of precipitate by centrifugation, the mixtures were then subjected to HPLC-ESI-HRMS analysis according to the procedure described above for BsfA precursor peptide.

### *In vitro* enzymatic assay

The assays for BsfK (total volume, 100 μL) were performed at 30°C for 5 h in 100 mM Tris–HCl buffer (pH 8.0) containing 5 mM MgCl_2_, 5 mM ATP, 200 μM BsfA (or the BsfA variants, BsfA core peptide) in the presence of 20 μM BsfB1 and 20 μM BsfK (or the BsfK variants). ATP was added last to initiate the reaction. The assays were quenched by adding an equal volume of CH_3_CN. After removal of precipitate by centrifugation, the mixtures were then subjected to HPLC-ESI-(HR)MS analysis according to the procedure described above for BsfA precursor peptide.

The assays for TF-BsfB2 (total volume, 100 μL) were performed at 30°C for 12 h in 100 mM K_2_HPO_4_ buffer (pH 7.5) containing 100 mM NaCl, 2 mM MgCl_2_, 2 mM ATP, 1 mM DTT, 100 μM BsfA in the presence of 20 μM BsfB1 and 20 μM TF-BsfB2. ATP was added last to initiate the reaction. After similar termination and centrifugation, the mixtures were then subjected to HPLC-ESI-(HR)MS analysis according to the procedure described above for BsfA precursor peptide.

## Results

### Identification of a conserved Ile(-2) residue in *Bradymonadales* derived lasso peptide precursors

**Figure 2:**
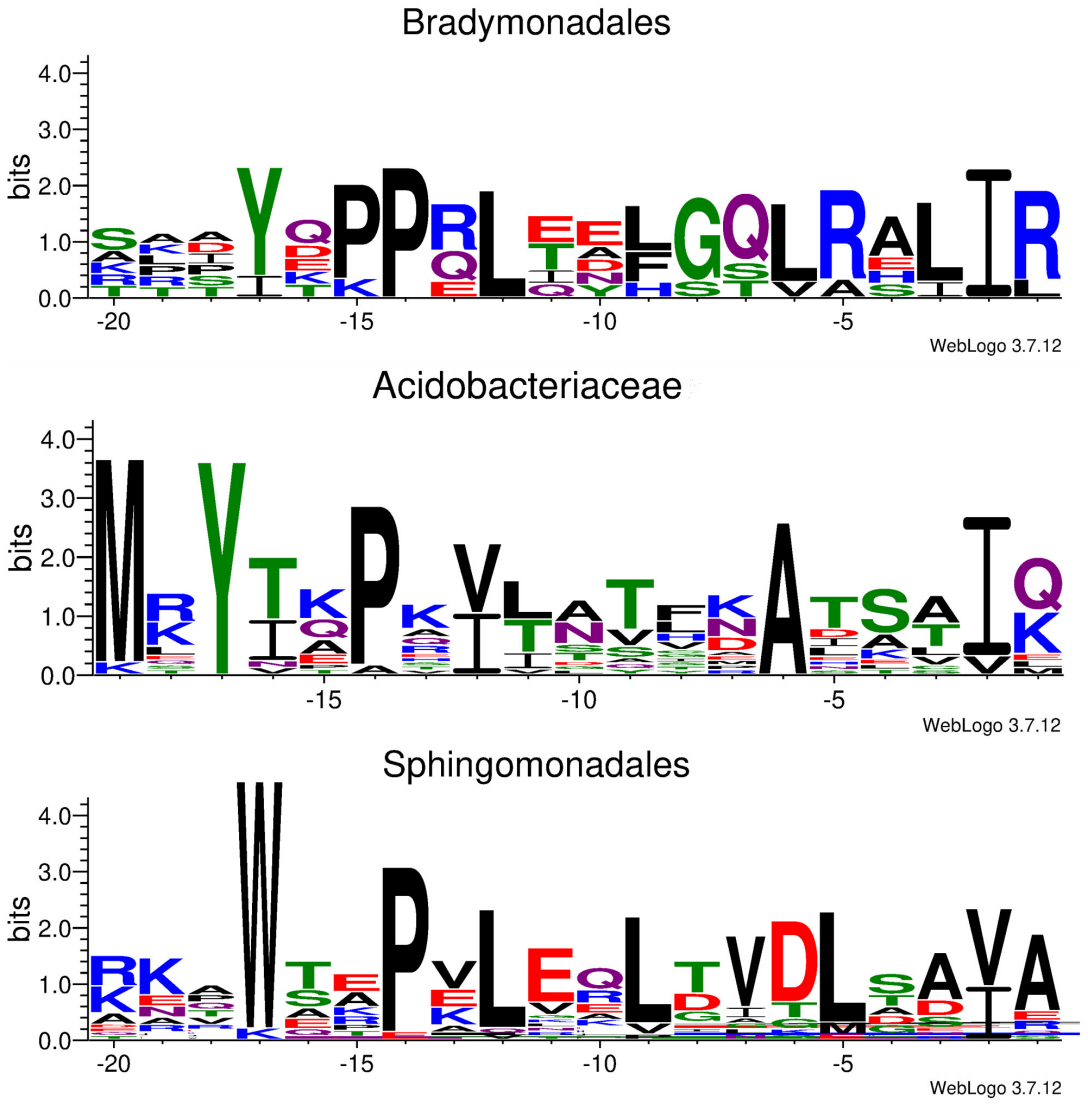
Conserved motif identified in Ile/Val(-2) containing leader peptides derived from *Bradymonadales*, *Acidobacteriaceae*, and *Sphingomonadales*, respectively.

Unfortunately, the *bsf* cluster is prone to be silent under laboratory conditions, and the *Bradymonas sediminis* FA350 strain is difficult to genetically manipulate. Thus, heterologous expression of the *bsf* cluster has been tried in other hosts. Briefly, the DNA fragment containing all the *bsf* genes and the flanking regions was removed from the genomic DNA by restriction digestion and further connected to the vector p15A via Red/ET recombination. The obtained plasmid (p15A-bsf) was then transformed into *E. coli* BL21(DE3), *E. coli* GB2005 and *Schlegelella brevitalea* DSM 7029, or conjugated into *Burkholderia gladioli* ATCC10248 and *Burkholderia thailandensis* E264, respectively, for heterologous expression in different culture media. However, no mass corresponding to the predicted lasso peptide has been observed. Moreover, several constitutive promotors such as P_Tn5-Km_, P_apra_ and the inducible promoter P_tet_ have been inserted upstream to *bsfC*, respectively, but the mature lasso peptide has never been detected. This results in difficulty to demarcate the boundary between the leader and the core peptide in BsfA. It was reported that the conserved Tyr(-17), Pro(-14), and Leu(-12) residues in leader peptides were required for the recognition by the B1 proteins (Chekan et al., 2019; Sumida et al., 2019). To identify the core peptide of BsfA, surveys of genomic data uncovered several similar lasso peptide BGCs in other *Bradymonadales*, including *Persicimonas caeni* YN101 (*pcy*), *Microvenator marinus* V1718 (*mmv*), *Lujinxingia litoralis* B210 (*llb*), *L. sediminis* SEH01 (*lss*) and *L. vulgaris* TMQ2 (*lvt*), et al. (Supplementary Figure S2). Compared with *bsf*, the *D* gene is lacking in *mmv* and the *N* genes are absent in the *llb*, *lss*, and *lvt* clusters. Sequence alignment of the precursor peptides showed a conserved YxxPxL (where x is any amino acid) motif (Figure 2; Supplementary Figure S2), indicating that these residues are always spanning positions (-17) to (-12) in leader peptides. Thus, the conserved Gly1 is at the *N*-terminus of the core peptide and it cyclizes with Asp8 to form the macrolactam ring. A threonine at the penultimate position of the leader region was hitherto found in all previously reported lasso peptide precursors and was shown to be a recognition element for effective enzymatic processing (Supplementary Figure S3; Pan et al., 2012; Hegemann et al., 2015). Nevertheless, our sequence alignment revealed that the penultimate residue in all lasso peptide precursors found in *Bradymonadales* spp. was an isoleucine, which was distinct from the known lasso peptides (Figure 2; Supplementary Figure S2).

### Characterization of the phosphorylation activity of BsfK in conjunction with BsfB1

**Figure 3:**
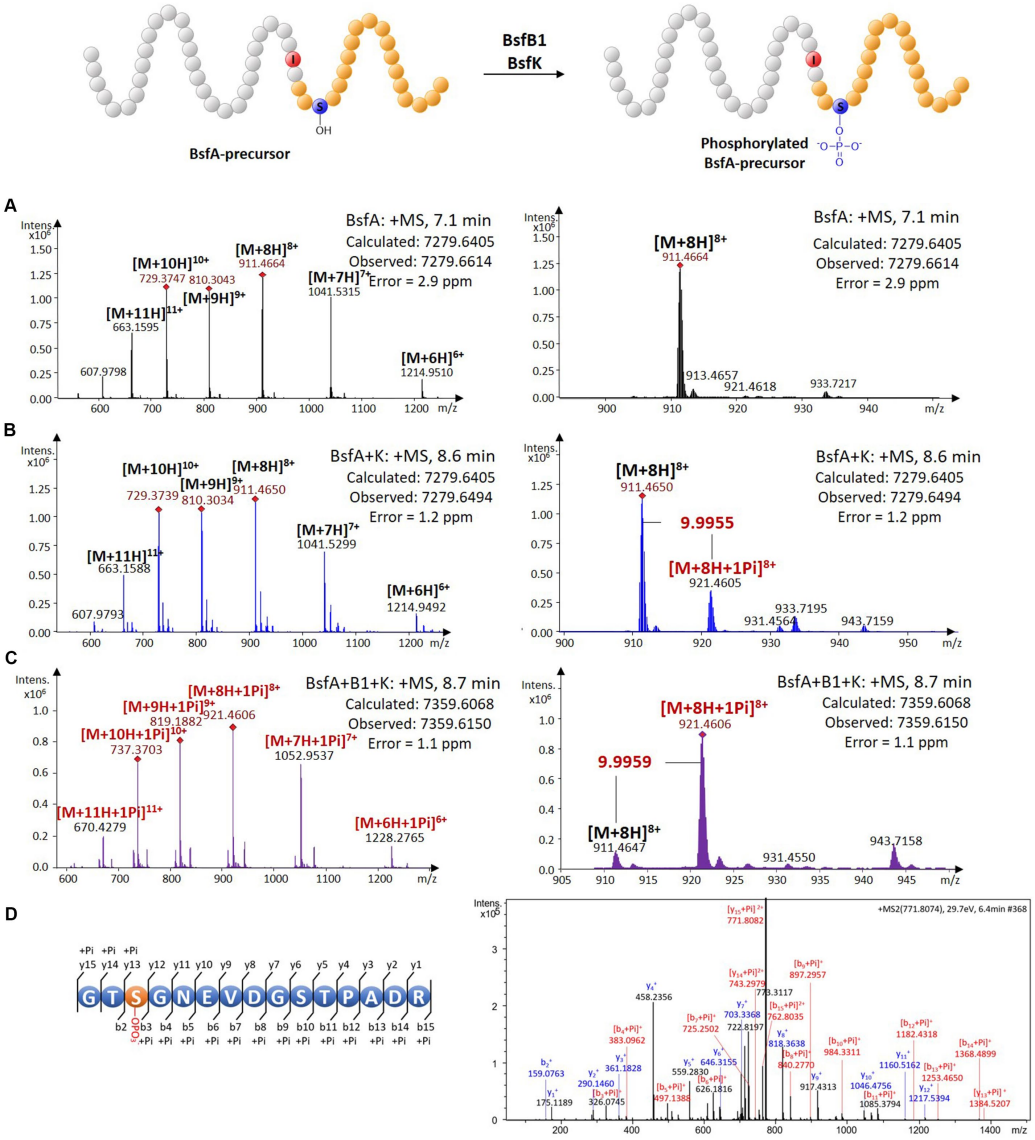
*In vivo* characterization of the phosphorylation activity on BsfA. **(A)** HRMS spectrum of unmodified BsfA. **(B)** HRMS spectrum of BsfA co-expressed with BsfK. **(C)** HRMS spectrum of BsfA co-expressed with BsfB1 and BsfK. **(D)** MS/MS fragmentation analysis of core peptide fragment after trypsin digestion.

A BLASTP search using the BsfK protein sequence as input revealed that it was a hypothetical protein with a very low identity (<35%) with histidine containing phospho carrier protein (HPr) kinases (Supplementary Figure S4). To validate the function of BsfK, the pRSFDuet-1 vector was used for the co-expression of *N*-terminally hexaHis-tagged BsfA (*BamH*I + *Hind*III) and untagged BsfK (*Nde*I + *Xho*I). After purification, high performance liquid chromatography-electrospray ionization-high resolution mass spectrometry (HPLC-ESI-HRMS) was used for the detection of PTMs in the *N*-terminally tagged BsfA. Apart from the dominant unmodified BsfA (calculated 7279.6405 Da, found 7279.6614 Da), a small component with a mass increase of 79.9640 Da was also detected, which was barely visible when *bsfK* was omitted (Figures 3A,B; Supplementary Figure S5). This increased mass was in line with a monophosphorylation of BsfA (calculated 79.9663 Da), indicating that BsfK was indeed a peptide kinase and catalyzed phosphorylation of the precursor peptide BsfA. Given that *B*. *sediminis* FA350 belongs to a novel order of *Deltaproteobacteria*, it was not surprising that BsfK showed a very low identity (<35%) with other proteins in the National Center for Biotechnology Information (NCBI) database.

Next, untagged BsfB1was introduced into the co-expression system above. In comparison with the BsfA and BsfK co-expression experiment (Figure 3B), the monophosphorylated BsfA was increased substantially and became dominant in the BsfB1-containing co-expression system (Figure 3C; Supplementary Figure S5). Unmodified BsfA was also observed, albeit in a minor amount. Evidently, BsfB1 greatly enhanced the phosphorylation activity of BsfK, just like in other cases RREs could increase the turnover rates for precursor epimerization and hydroxylation (Feng et al., 2020; Zhang and Seyedsayamdost, 2020).

Phosphorylation is a very common PTM in RiPP biosynthesis, which usually occurs at the hydroxyl groups of Ser or Thr residues (Montalbán-López et al., 2021). The phosphorylations previously reported in lasso peptides were introduced into the precursors prior to leader excision and core macrolactamization, and were all located at the *C*-terminal Ser residues of the precursors (Zhu et al., 2016b,c; Zyubko et al., 2019). This cannot occur in BsfA with a *C*-terminal aspartate residue. Additionally, the identities between BsfK and the known lasso peptide kinases are relatively low (<21%), indicative of a distinct phosphorylated position from the known lasso peptides. Trypsin digestion and subsequent tandem MS analysis of phosphorylated precursor peptide pointed to the Ser3 residue as the modification site by BsfK (Figure 3D; Supplementary Table S1). This was further confirmed by co-expression experiments of site-directed mutations as BsfA S3A was principally unmodified despite a low production yield, whereas T2A, S10A and T11A substitutions displayed a majority of monophosphorylated peptide, just like the native BsfA (Supplementary Figure S6). It is reasonable to speculate that similar phosphorylations also occurred at the same position in other *Bradymonadales* derived clusters, as Ser3 in BsfA is conserved among all the precursor peptides except for LvtA, which contains a threonine instead of serine at this position (Supplementary Figure S2).

To expand the repertoire of BsfK beyond Ser phosphorylation, Ser3 was mutated to other hydroxyl containing residues (i.e., Thr and Tyr). It was observed that while BsfA S3T was phosphorylated efficiently, no phosphorylation was observed with BsfA S3Y, surmising that BsfK is somewhat substrate tolerant (Supplementary Figure S7; Supplementary Table S2). This finding makes it likely that the Thr3 residue in the LvtA precursor could also be phosphorylated by LvtK. In addition, the kinase BsfK exhibits strict regioselectivity for the third residue of the BsfA core peptide, since the phosphorylation of Thr2 has never been detected.

We heterologously expressed the kinase BsfK and BsfB1 to further certify their functions (Supplementary Figure S8). After purification, the obtained *N*-terminally hexaHis-tagged BsfK was characterized *in vitro* in the presence of the BsfA-precursor, ATP and magnesium chloride. In accordance with the *in vivo* characterization, BsfK phosphorylated the precursor peptide in a very low yield, whereas no conversion was observed in the absence of ATP, magnesium chloride, or with heat-treated BsfK (Supplementary Figure S9). This process was greatly accelerated by adding BsfB1, suggesting that the RRE protein plays an important role in phosphorylation. In addition to ATP, other NTPs are also able to act as phosphate donors for the precursor peptide phosphorylation (Supplementary Figure S9).

Sequence alignment of the *Bradymonadales* derived lasso peptide kinases and the previously characterized lasso peptide precursor kinases reveals a highly conversed motif (i.e., H148-G163-G166-G168-K169-S170-D186-D187 in BsfK; Supplementary Figure S10). The His residue is proposed to stabilize the last Asp, the Lys and the three glycine residues are considered to facilitate the binding of ATP, whereas the Ser and the conjugate Asp residues directly coordinate a magnesium ion or interact with this ion through a water intermediary (Allen et al., 2003; Zhu et al., 2016c). The importance of the corresponding residues in BsfK were validated by alanine substitutions. H148A, G163A and D186A exchanges greatly suppress the solubility of BsfK, indicative of their prominent roles in protein folding, while other variants show little influence on the expression of BsfK (Supplementary Figure S8). *In vitro* characterization of the variants revealed that G166A and G168A have almost the same catalytic activity compared with wild type BsfK, whereas the activities of other variants are diminished in varying degrees (Supplementary Figure S11). These results point out the significance of H148, G163, K169, S170, D186 and D187 for BsfK catalysis, just like the known lasso peptide kinases.

### Validation of the role of the Ile(-2) residue in BsfA in lasso peptide processing

**Figure 4:**
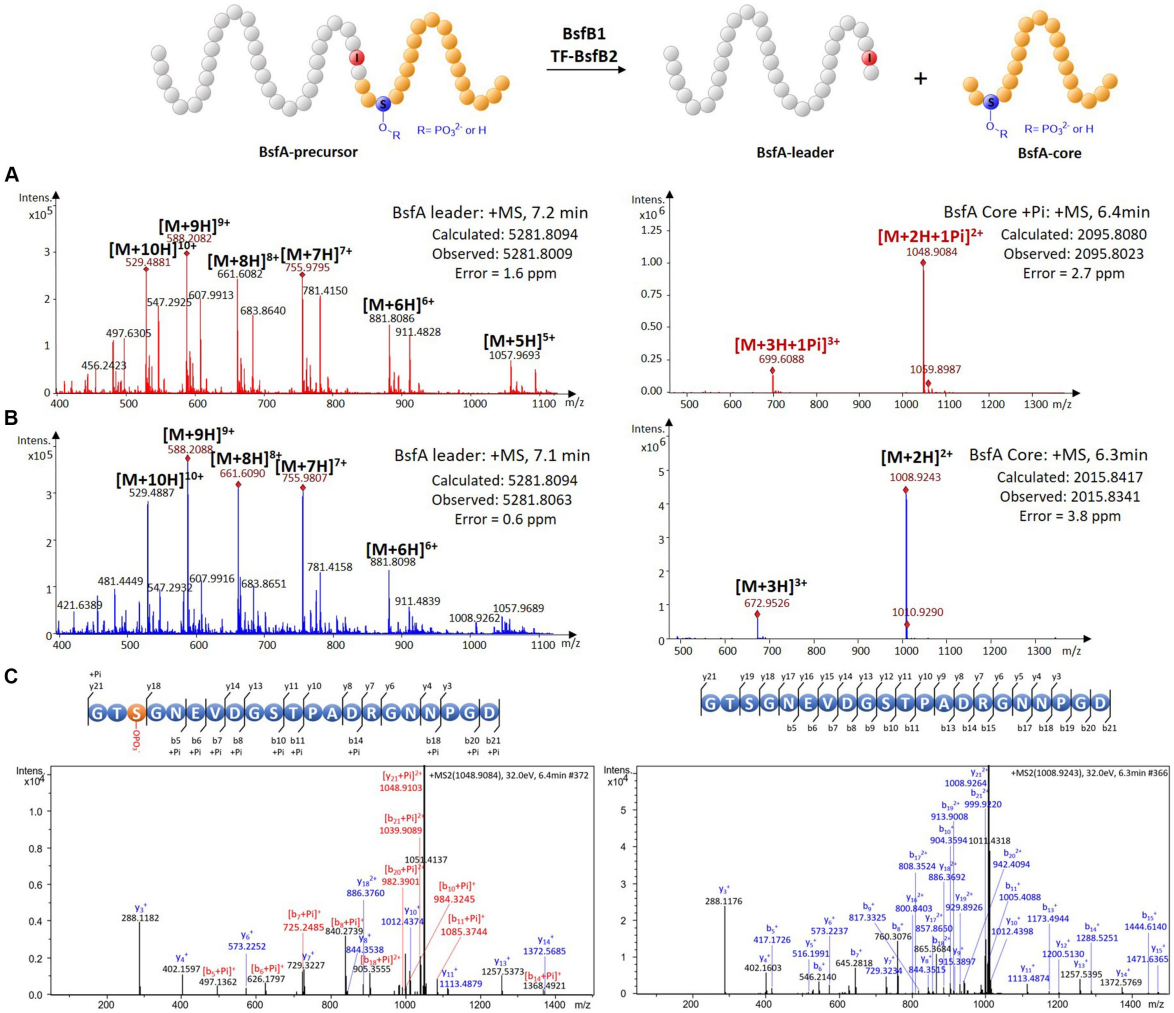
*In vitro* characterization of the peptidase activity of BsfB2. **(A)** HRMS spectra of leader and core peptides released through BsfB2 treatment of phosphorylated BsfA precursor. **(B)** HRMS spectra of leader and core peptides released through BsfB2 treatment of unmodified BsfA precursor. **(C)** MS/MS fragmentation analysis of (phosphorylated) core peptide fragments.

Since the *bsf* cluster is silent under laboratory conditions, we attempted to characterize the protease BsfB2 to further affirm the demarcation between the leader and the core peptide. The cold shock-induced expression vector pCold TF was used to express the BsfB2 carrying an *N*-terminal trigger factor (TF) tag (Supplementary Figure S8). HPLC-ESI-HRMS analysis of the assay containing phosphorylated BsfA-precursor, ATP, magnesium chloride, BsfB1 and TF-BsfB2 revealed a dramatic decrease of the precursor peptide and the emergence of two novel peaks with the masses 2095.8023 and 5281.8009 Da compared with the negative controls omitting BsfB1 or BsfB2 (Figure 4A; Supplementary Figure S12). The former mass is identical to the phosphorylated core peptide consisting of the *C*-terminal 21 amino acids of the precursor (calculated: 2095.8080 Da), which was confirmed by MS/MS fragmentation analysis (Figure 4C; Supplementary Table S3). The latter mass is equal to the *N*-terminal methionine excised and hexaHis-tagged leader peptide (calculated: 5281.8094 Da). A similar result was obtained when unphosphorylated precursor peptide was used instead of phosphorylated precursor. The mass of unphosphorylated BsfA core peptide (2015.8341 Da, calculated, 2015.8417 Da) was detected and its identify was further verified by MS/MS analysis (Figures 4B,C; Supplementary Table S4). These results indisputably proved that the penultimate position of the leader peptide is an atypical isoleucine residue rather than threonine. The necessity of BsfB1 for precursor peptide phosphorylation and leader peptide cleavage irrefutably illustrated its dual functions in lasso peptide maturation. Moreover, no phosphorylated peptide was generated when the core peptide was utilized as the substrate of BsfK (Supplementary Figure S13), which is attributable to the fact that neither the kinase nor the RRE did recognize the core peptide as substrate.

To the best of our knowledge, BsfA is the first experimentally validated, naturally occurring B2 protein substrate to include an Ile residue at the penultimate position of the leader peptide, while several previously reported lasso peptide precursors carrying T(-2)I substitutions were still accepted as substrates from the processing machinery, albeit with lower turnover (Pan et al., 2012; Hegemann et al., 2013, 2014). In addition, a putative Ile(-2) residue was found in a lasso peptide BGC from *Acidobacteriaceae bacterium* TAA 166 (Tietz et al., 2017), and a number of bioinformatically predicted lasso peptide precursors with putative Ile/Val/Ala-2 residues were reported during the submission of this manuscript (Kretsch et al., 2023). Besides, our *in-silico* analyses of *Acidobacteriaceae* bacteria also helped to identify an array of other lasso peptide precursors containing penultimate isoleucine or valine residues in their leader peptide precursors (Figure 2; Supplementary Figure S14), suggesting that the leader peptide Ile/Val(-2) residue may be widely distributed in *Acidobacteriaceae* derived lasso peptides. In addition to *Bradymonadales* and *Acidobacteriaceae*, leader peptide Ile/Val(-2) residue is also observed in a list of *Sphingomonadales* derived lasso peptide precursors (Figure 2; Supplementary Figure S14). These analyses strongly indicated that the leader peptide Ile/Val(-2) residues are rare but not uncommon in lasso peptide precursors.

### The effects of Ile(-2) and the conserved YxxPxL motif on phosphorylation and leader peptide removal

**Figure 5:**
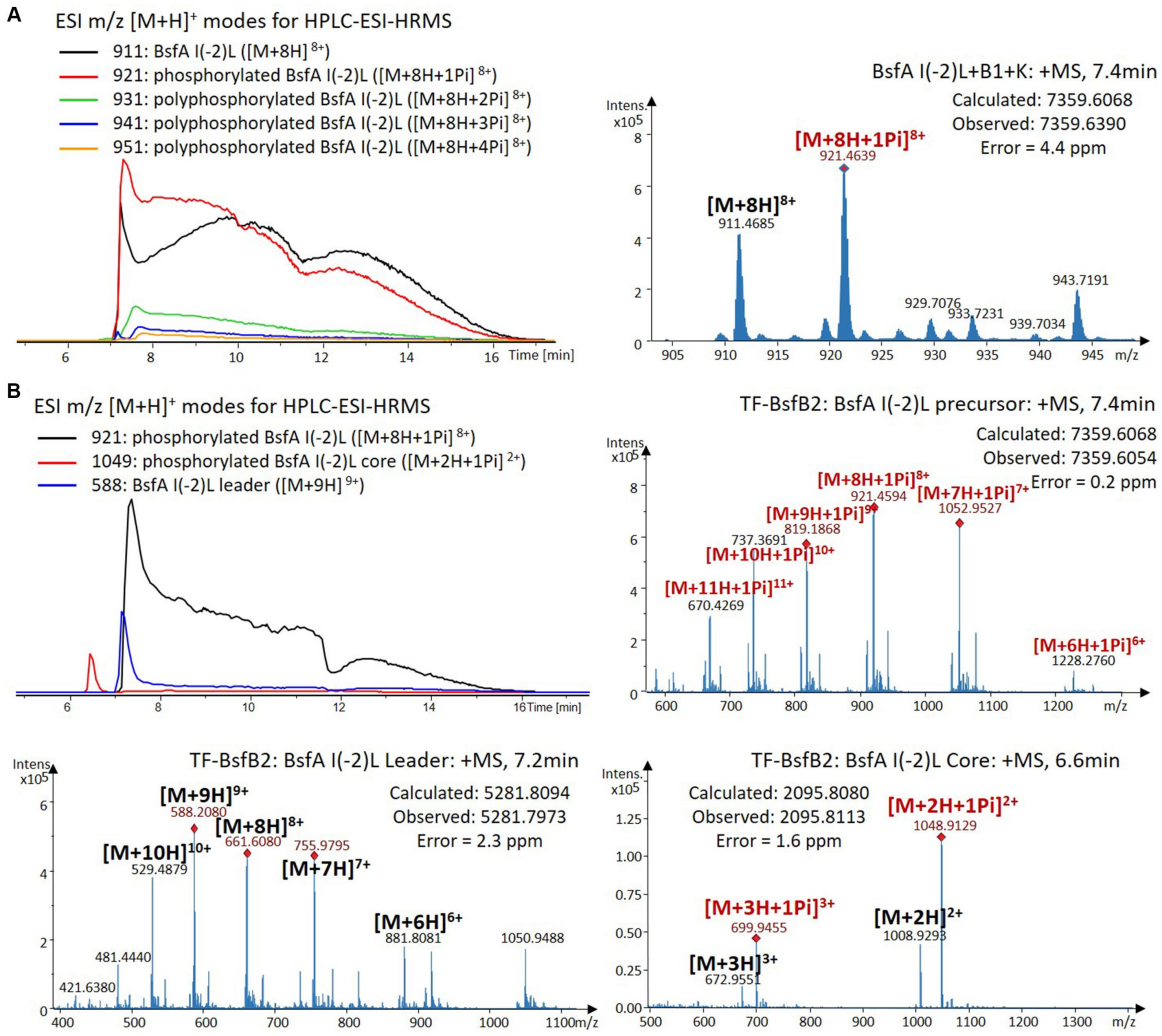
**(A)** HPLC-ESI-HRMS analysis of phosphorylated BsfA I(-2)L purified from co-expression with BsfB1 and BsfK. **(B)** *In vitro* characterization of TF-BsfB2 with phosphorylated BsfA I(-2)L.

**Figure 6:**
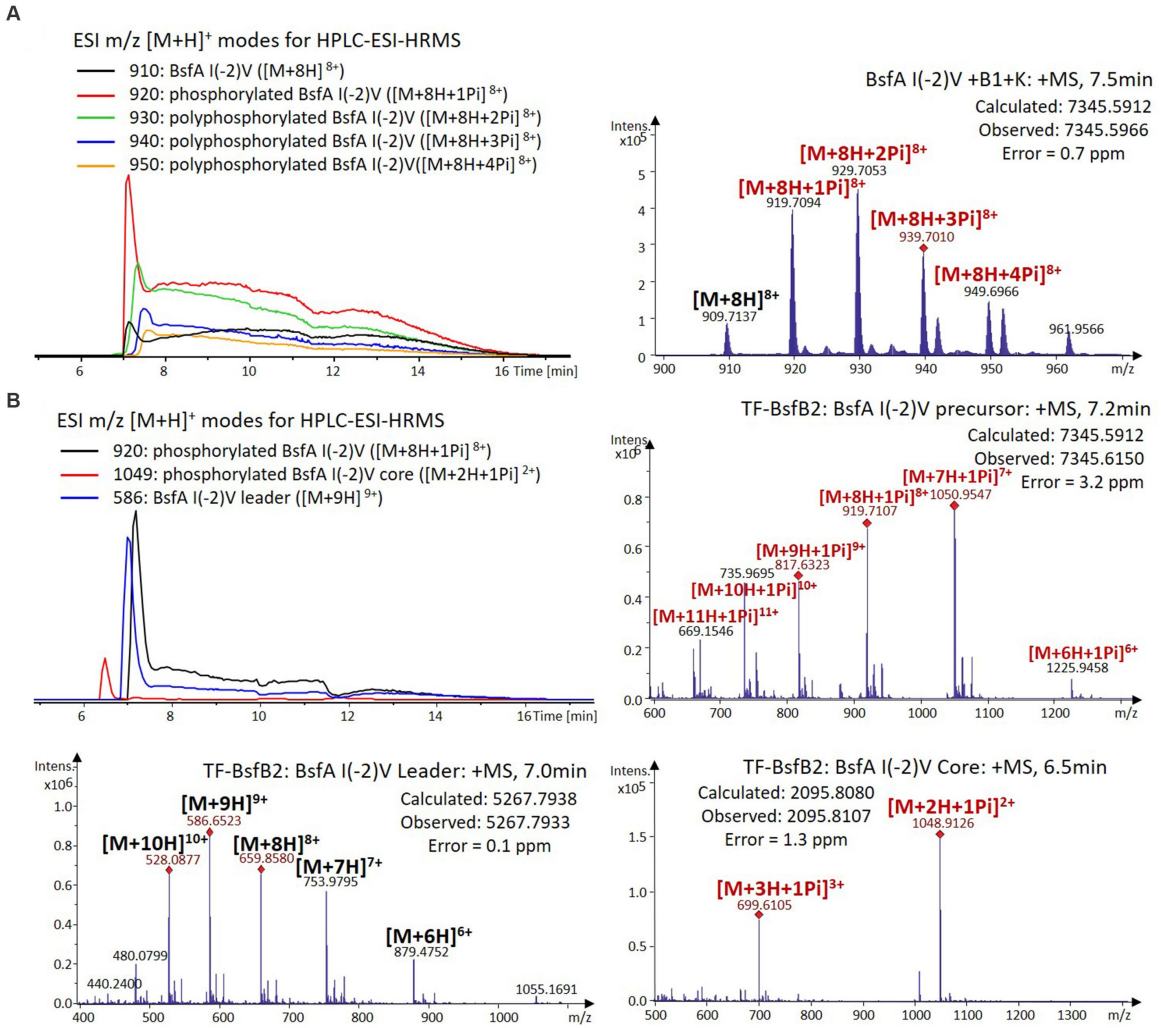
**(A)** HPLC-ESI-HRMS analysis of phosphorylated BsfA I(-2)V purified from co-expression with BsfB1 and BsfK. **(B)** *In vitro* characterization of TF-BsfB2 with phosphorylated BsfA I(-2)V.

**Figure 7:**
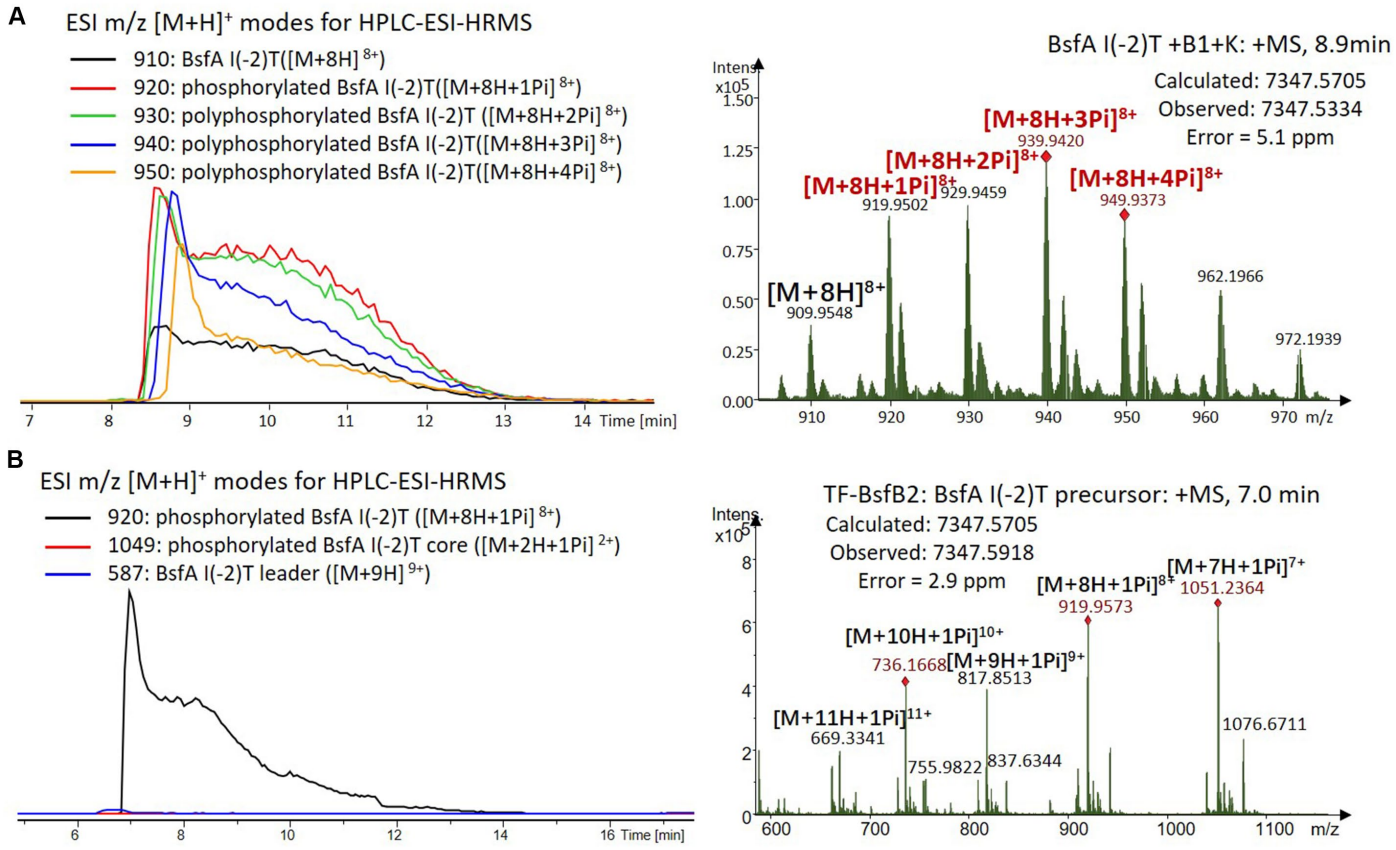
**(A)** HPLC-ESI-HRMS analysis of phosphorylated BsfA I(-2)T purified from co-expression with BsfB1 and BsfK. **(B)** *In vitro* characterization of TF-BsfB2 with phosphorylated BsfA I(-2)T.

**Figure 8:**
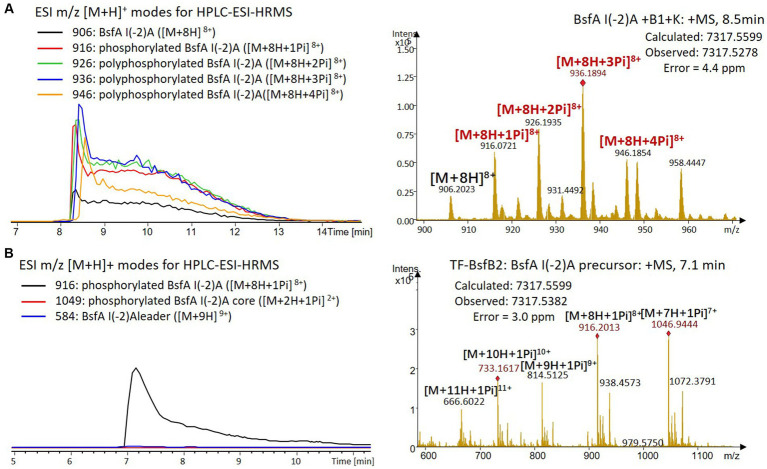
**(A)** HPLC-ESI-HRMS analysis of phosphorylated BsfA I(-2)A purified from co-expression with BsfB1 and BsfK. **(B)** *In vitro* characterization of TF-BsfB2 with phosphorylated BsfA I(-2)A.

We were curious to know whether the leader peptide Ile(-2) residue in BsfA also plays a role in phosphorylation and leader peptide removal. This residue was targeted for replacement with other amino acids. Ile(-2) was first exchanged with the congener leucine residue in BsfA, which was then co-expressed with BsfB1 and BsfK. HPLC-ESI-HRMS analysis of the BsfA variant after purification and desalting revealed that only part of the precursor peptide was phosphorylated (Figure 5A), compared with the wild type BsfA where almost all the precursor was phosphorylated (Supplementary Figure S5). Most of the I(-2)V precursor peptide was phosphorylated, while in addition to the major monophosphorylated precursor, we detected a considerable amount of polyphosphorylated precursor peptides (Figure 6A), which were less detectable in wild type BsfA. Assays with the I(-2)L, I(-2)T, and I(-2)A variants of BsfA yielded similar results to the assay with BsfA I(-2)V (Figures 7A, 8A), implying that the penultimate residue in the leader peptide has a great influence on the degree of phosphorylation. Tandem MS analysis of BsfA I(-2)A after trypsin digestion identified Ser3 as the polyphosphorylated residue, which was corroborated by the observation that the BsfA I(-2A)&S3A variant could not be phosphorylated (Supplementary Figure S15; Supplementary Table S5). Polyphosphorylations on *C*-terminal serine residues were reported for ThcoK and SyanK (Zhu et al., 2016b), whereas our result pointed to (poly)phosphorylation on a ring residue of the lasso peptide.

The BsfA Ile(-2) substitution variants were furthermore utilized for the characterization of TF-BsfB2 activity. In contrast to the wild type BsfA that was cleaved almost completely, I(-2)T as well as I(-2)A exchanges completely hindered the removal of the leader peptide even in the presence of BsfB1 (Figures 7B, 8B). In assays using the I(-2)L and I(-2)V exchange variants, barely any leader cleavage was observed either (Figures 5B, 6B). Very recently, RRE domains in lasso peptide biosynthesis were shown not only to deliver the precursor peptide to the leader peptidase, but also to partially compose the elusive S2 proteolytic pocket that binds the penultimate residue of the leader peptides. A critical tyrosine residue (Tyr33 in FusE) in these RRE domains is suspected to directly engage Thr(-2), perhaps via side chain H-bonding (Alfi et al., 2022; Kretsch et al., 2023). However, this is unlikely for the case of BsfB1, as the Ile(-2) residue does not contain polar groups for such kind of interactions. Sequence alignment reveals that BsfB1 also contains this tyrosine residue (Tyr37 in BsfB1), but this residue is not conserved in *Bradymonadales* derived B1 proteins (Supplementary Figure S16). The interaction between BsfB1 and the penultimate isoleucine residue in BsfA remains largely enigmatic and is worthy of further investigation. Thus, we speculate that the penultimate isoleucine residue in BsfA affects both BsfB1 and BsfB2, and that these proteins exhibit a new substrate selectivity for leader peptide binding and excision.

Ala-replacements of Tyr(-17), Pro(-14), and Leu(-12) were also performed and tested for their effects on phosphorylation and leader peptide excision. The Y(-17)A and L(-12)A exchanges did not have any effect on the phosphorylation of BsfA, as the masses of unphosphorylated precursors are barely detected in HRMS analyses. Nevertheless, the P(-14)A variant precipitates immediately after addition to the BsfK assay, and no mass of the precursor is observed. Thus, double (Y(-17)A&P(-14)A) and triple (Y(-17)A&P(-14)A&L(-12)A) exchange variants of BsfA were generated. Surprisingly, neither the double nor the triple Ala substitution affects precursor phosphorylation (Supplementary Figure S17). Moreover, subsequent characterization of BsfB2 revealed that for all single and double substitution variants, the leader peptide were excised effectively, including the unstable P(-14)A variant. In contrast, the triple substitution variant was only partially cleaved (Supplementary Figure S18). We surmise that other residues in BsfA leader peptide in addition to Tyr(-17), Pro(-14), and Leu(-12) may interact with BsfB1 as well, whereas the possibility that protein–protein interactions of BsfB1/BsfK or BsfB1/BsfB2 facilitate the enzymatic activities needs to be further investigated in the future.

## Discussion

Our results demonstrate that the kinase BsfK specifically catalyzes the phosphorylation of the Ser3 residue in BsfA, while BsfB1 performs dual functions to accelerate the post-translational phosphorylation and to assist BsfB2 in leader peptide removal. Moreover, it is noteworthy that the penultimate residue in the leader peptide is isoleucine rather than the conserved threonine in all other so far investigated lasso peptide precursors. This Ile(-2) residue has a profound effect on the monophosphorylation of Ser3 and leader peptide removal, both of which are decreased in Ile(-2) substitution variants. The presence of a penultimate Ile/Val residue was also observed in putative lasso peptide precursors from *Acidobacteriaceae* and *Sphingomonadales*.

PadeB2 from *Paenibacillus dendritiformis* C454, ThcoB2 from *Thermobacillus composti* KWC4, BaceB2 from *Bacillus cereus* VD115, PapoB2 from *Paenibacillus polymyxa* CR1, SyanB2 from *Sphingobium yanoikuyae* ATCC 51230 and PsmB2 from *Bacillus pseudomycoides* DSM 12442 are the only B2 proteins reported to be involved in the biosynthesis of phosphorylated lasso peptides (Zhu et al., 2016b,c; Zyubko et al., 2019). Phylogenetically, B2 proteins from Ile/Val(-2) residue containing BGCs fall into clades close to the PadeB2 group but far from other reported B2 proteins (Supplementary Figure S19). Considering that all the Ile/Val(-2) residue containing lasso peptide BGCs unveiled by us also encode a kinase or phosphotransferase, we hypothesize that these B2 proteins and the PadeB2 group stem from a common ancestor.

The coorperation of lasso peptide B1 and B2 proteins is well known for leader peptide removal (Zhu et al., 2016a; Hegemann et al., 2018; DiCaprio et al., 2019; Koos and Link, 2019), which was also attested by our *in vitro* characterization of BsfB1 and BsfB2. In addition, the enhancement of phosphorylation in the presence of BsfB1 is probably owed to the recruitment with BsfK for leader peptide dependent PTM. Since only a few macrolactam synthetases have been characterized *in vitro* (Duquesne et al., 2007; Yan et al., 2012; Li et al., 2015; DiCaprio et al., 2019; Koos and Link, 2019), and there is no evidence for the function and timing of BsfN, further studies on these enzymes are currently underway in our laboratory.

## Data availability statement

The datasets presented in this study can be found in online repositories. The names of the repository/repositories and accession number(s) can be found in the article/Supplementary material.

## Author contributions

YD carried out the experiments. YD, WN, and LP performed the *in-silico* analyses. D-SM and Z-JD assisted with data analysis and manuscript preparation. GZ and YD analyzed the data and wrote the manuscript. GZ, XB, and YZ designed the work. All authors contributed to the article and approved the submitted version.

## Funding

This work was supported by the National Natural Science Foundation of China (21907057 and 32170038), the Natural Science Foundation of Jiangsu Province, China (BK20190201), the Future Plan for Young Scholars, and the Fundamental Research Funds (2019GN032) of Shandong University.

## Conflict of interest

The authors declare that the research was conducted in the absence of any commercial or financial relationships that could be construed as a potential conflict of interest.

## Publisher’s note

All claims expressed in this article are solely those of the authors and do not necessarily represent those of their affiliated organizations, or those of the publisher, the editors and the reviewers. Any product that may be evaluated in this article, or claim that may be made by its manufacturer, is not guaranteed or endorsed by the publisher.

## Footnotes

^1^ http://nocardia.nih.go.jp/fp4/

^2^ http://blast.ncbi.nlm.nih.gov/Blast.cgi

^3^ http://www.bioinformatics.org/strap/

^4^ https://weblogo.threeplusone.com/create.cgi
