# Supplementary material for: Leader peptide removal in lasso peptide biosynthesis based on penultimate isoleucine residue
Source: Front Microbiol. 2023 Jul 10;14:1181125. doi: 10.3389/fmicb.2023.1181125 (PMC10368454; doi:10.3389/fmicb.2023.1181125)
Supplement: Supplementary file 1 [file Data_Sheet_1.PDF]

Supplementary Material

Leader peptide removal in lasso peptide biosynthesis based on penultimate isoleucine residue

Yuwei Duan,[1] Weijing Niu,[1] Linlin Pang,[1] Da-Shuai Mu,[1,4] Zong-Jun Du,[1,4] Youming Zhang,[1,3]* Xiaoying Bian[1]* and Guannan Zhong[1,2]*

*** Correspondence:** Guannan Zhong: zhonggn@sdu.edu.cn, Xiaoying Bian: bianxiaoying@sdu.edu.cn, Youming Zhang: zhangyouming@sdu.edu.cn

Supplementary Figures

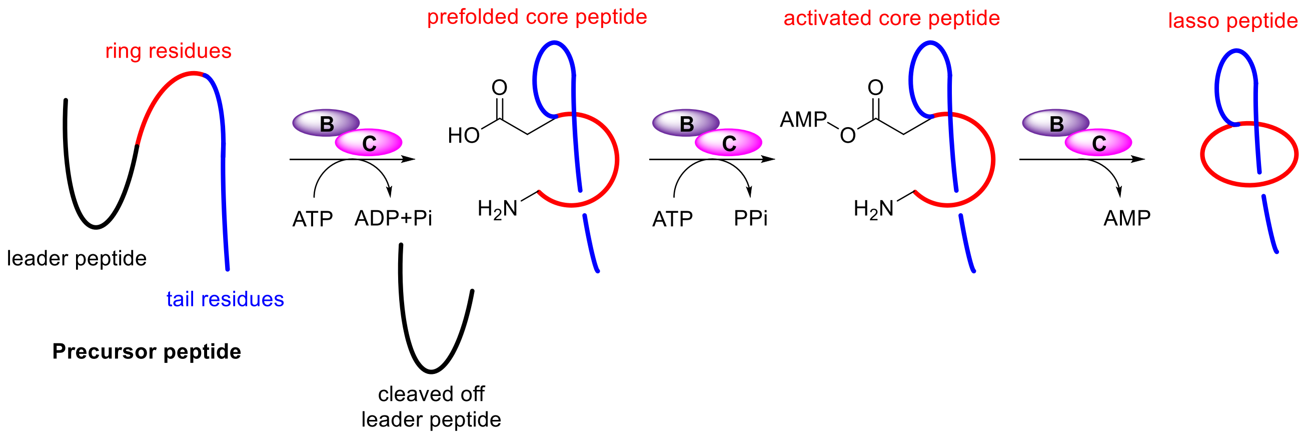

Supplementary Figure 1. Typical biosynthetic pathway of lasso peptides.

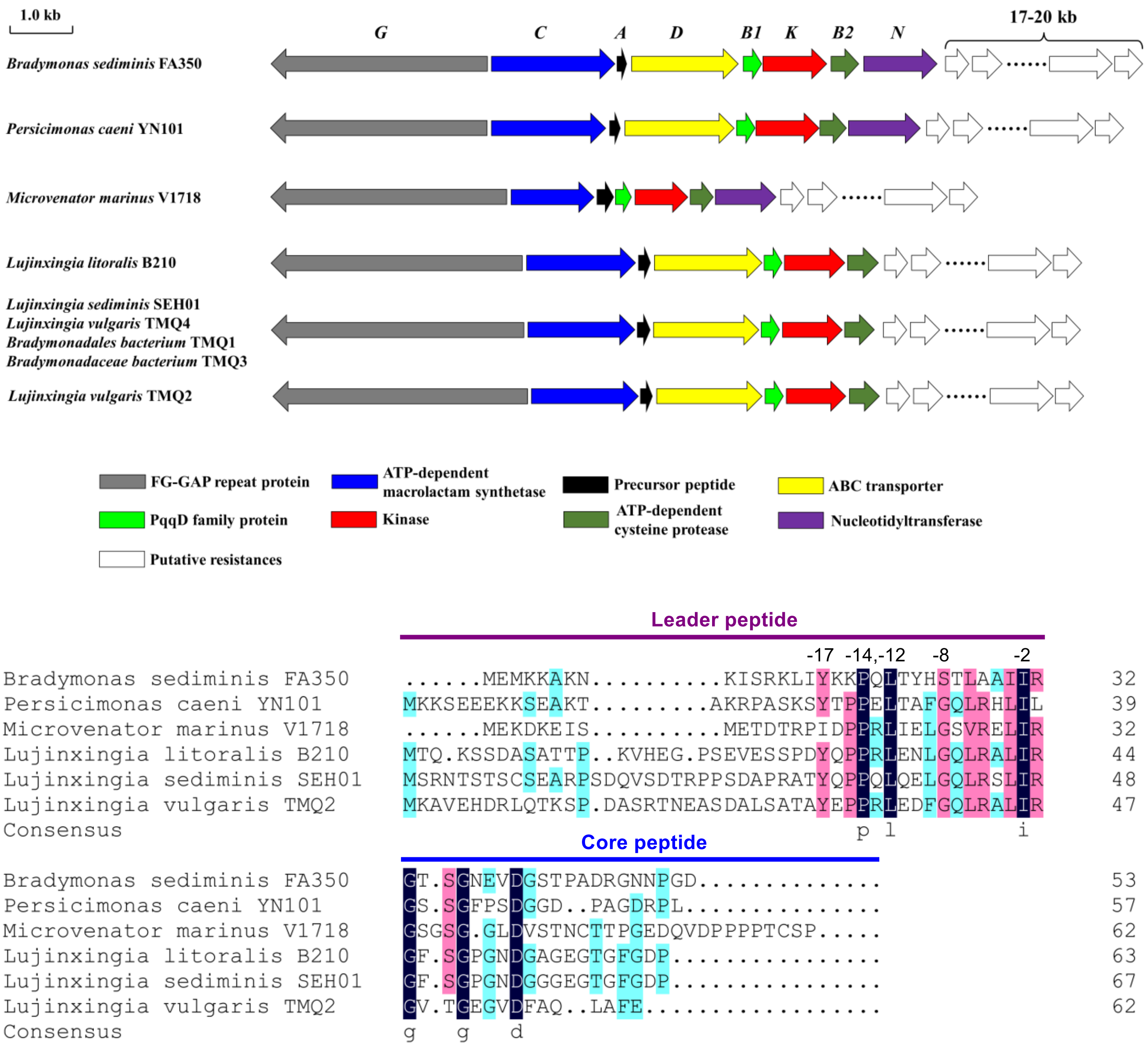

Supplementary Figure 2. Lasso peptide BGCs from different *Bradymonadales* and sequence alignment of the precursor peptides.

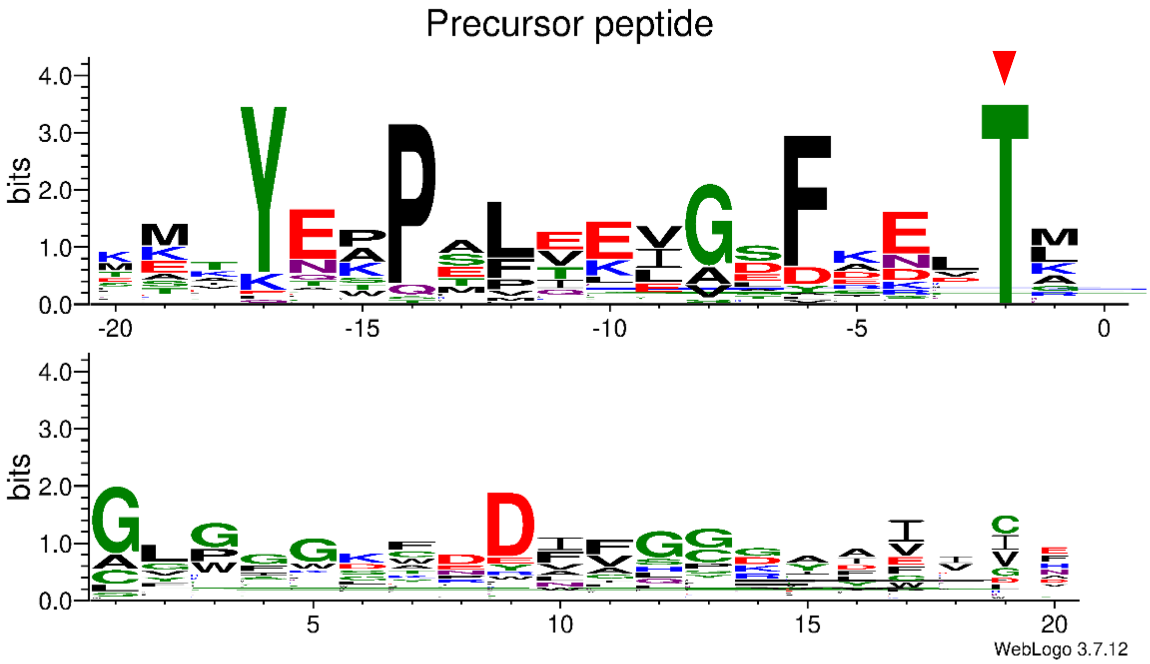

Supplementary Figure 3. Conserved motif identified in lasso peptide precursors using sequences from the 45 functional precursors as query (please see supporting information 2 for the sequences). The conserved Thr(-2) residue is indicated by red triangle.

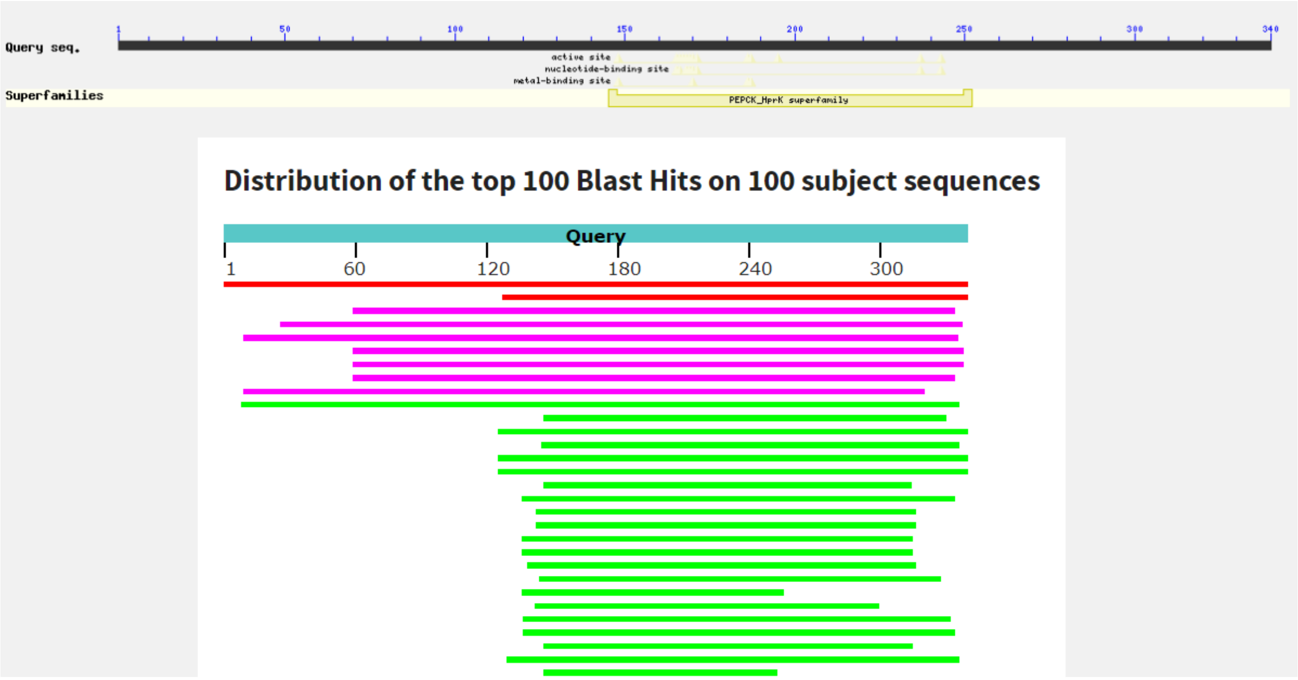

Supplementary Figure 4. Protein BLAST result for BsfK.

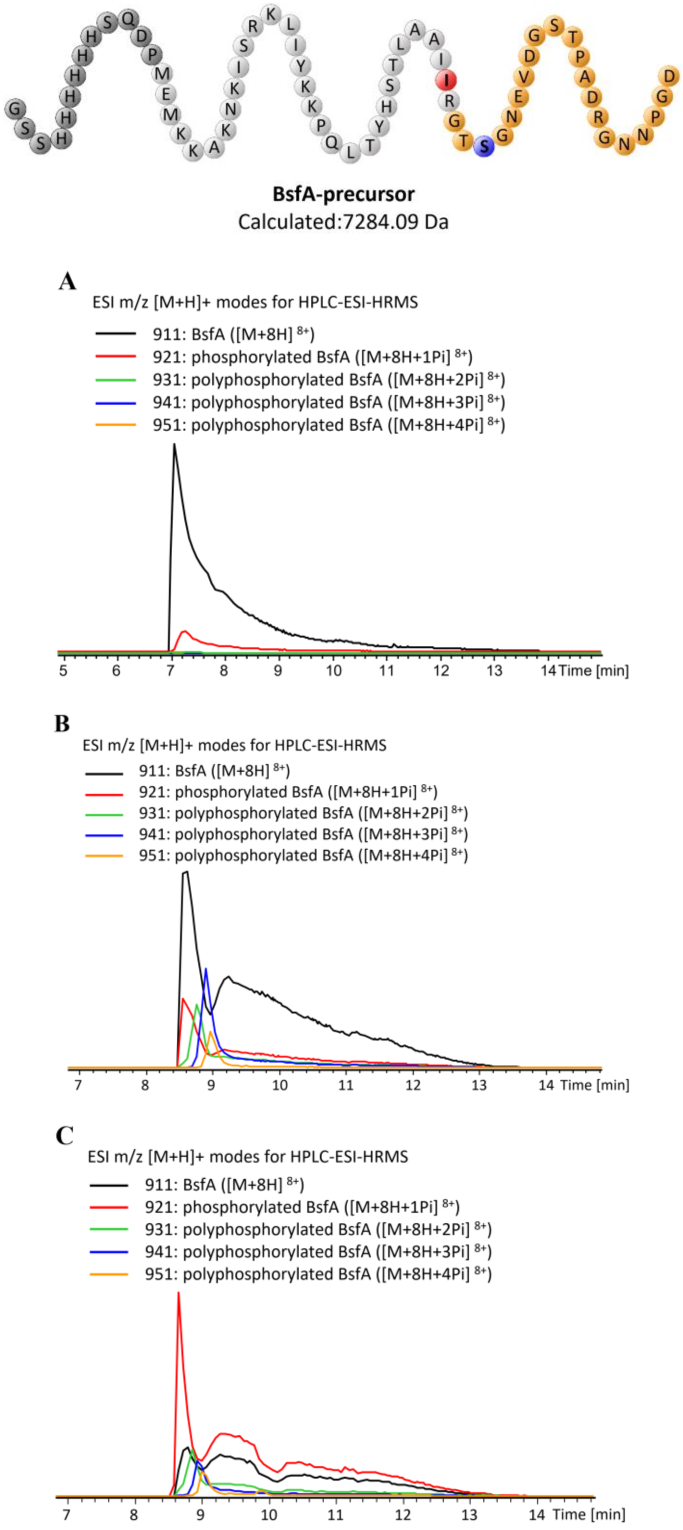

Supplementary Figure 5. HPLC-ESI-HRMS analyses of precursor peptides purified from (co-)expressions. (**A**) HPLC spectrum of purified BsfA. (**B**) HPLC spectrum of BsfA co-expressed with BsfK. (**C**) HPLC spectrum of BsfA co-expressed with BsfB1 and BsfK.

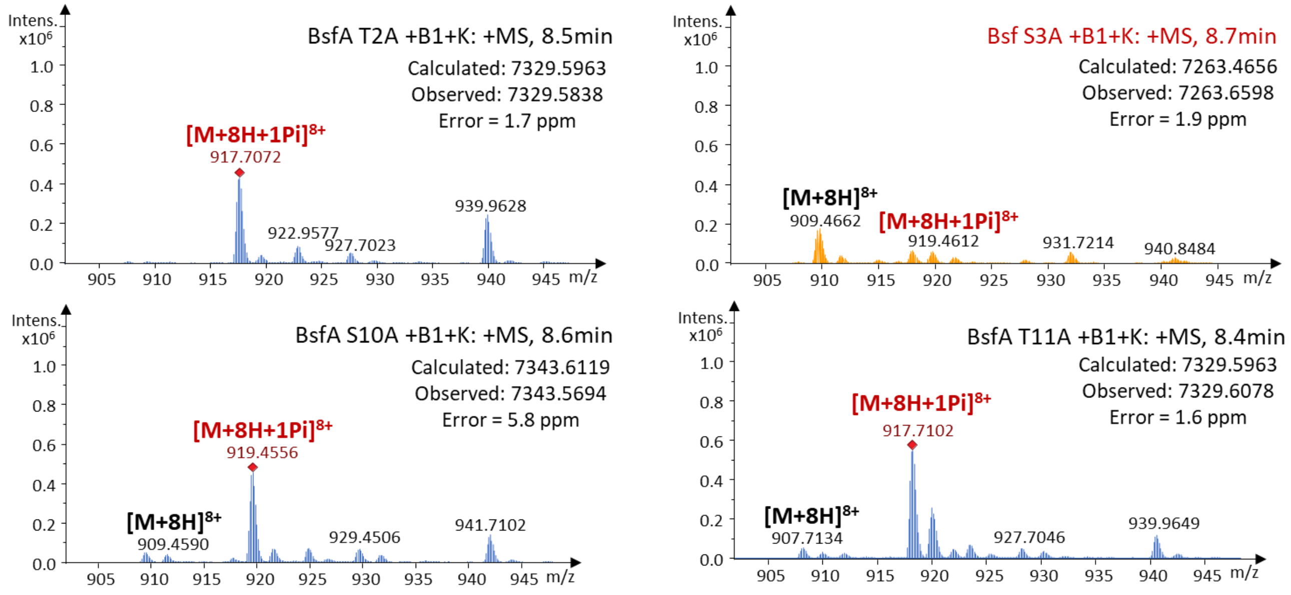

Supplementary Figure 6. HPLC-ESI-HRMS analyses of the Ala-replacements of BsfA Thr2, Ser3, Ser10 and Thr11 purified from co-expressions with BsfB1 and BsfK. The variants of T2A, S10A and T11A displayed a majority of monophosphorylated peptide, just like the wild type BsfA, whereas S3A was principally unmodified. The mass of $[M+8H+1Pi]^{8+}$ in the S3A variant may result from a non-specific monophosphorylation by a kinase located outside of the *bsf* cluster.

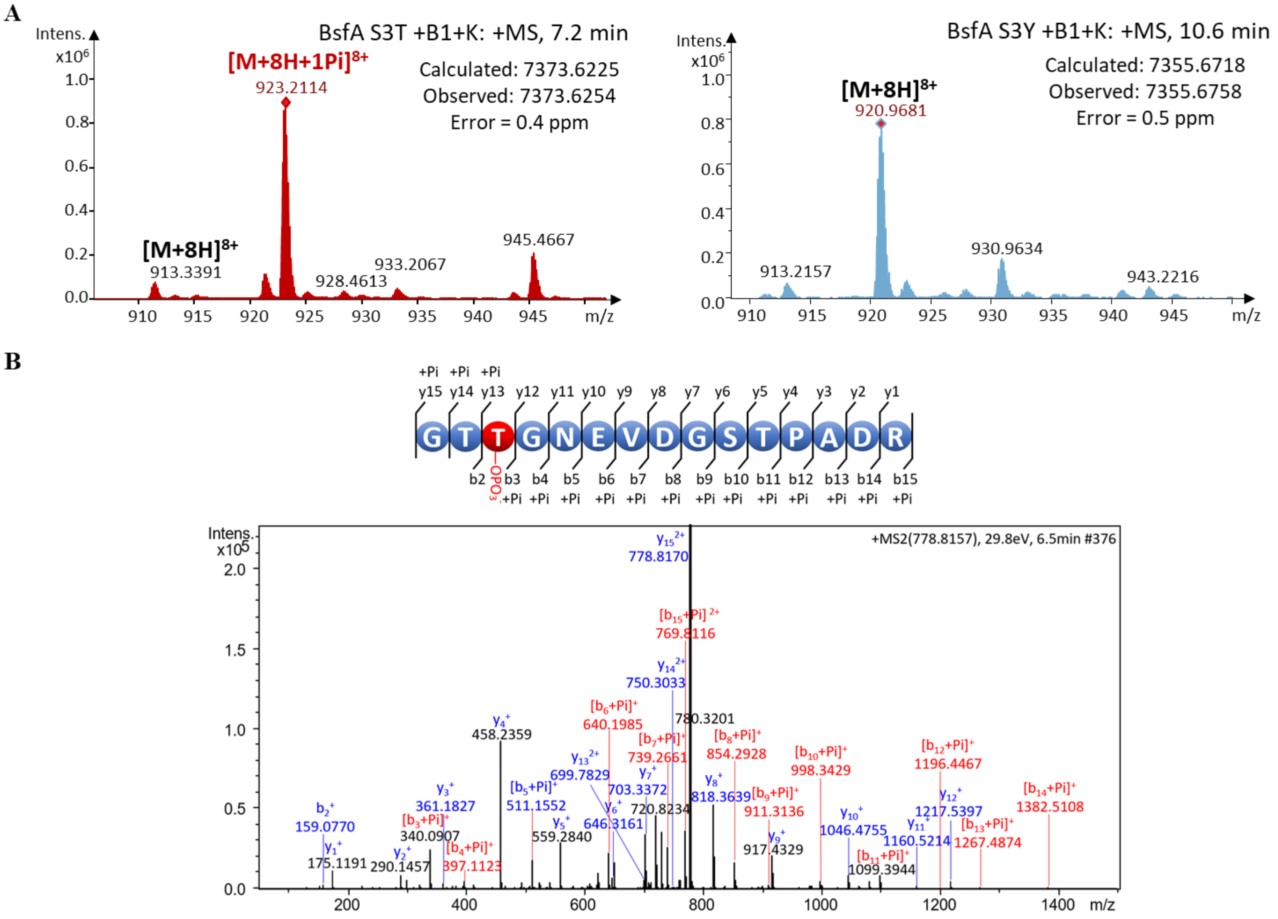

Supplementary Figure 7. Characterization of BsfK with S3T and S3Y precursor variants. (**A**) HPLC-ESI-HRMS analyses of BsfA S3T and S3Y purified from co-expressions with BsfB1 and BsfK. The variant of S3T displayed a majority of monophosphorylated peptide, just like the wild type BsfA, whereas S3Y was principally unmodified. The mass of $[M+8H+1Pi]^{8+}$ in the S3Y variant may result from a non-specific monophosphorylation by a kinase located outside of the *bsf* cluster. (**B**) MS/MS fragmentation analysis of the BsfA S3T core peptide fragment after trypsin digestion.

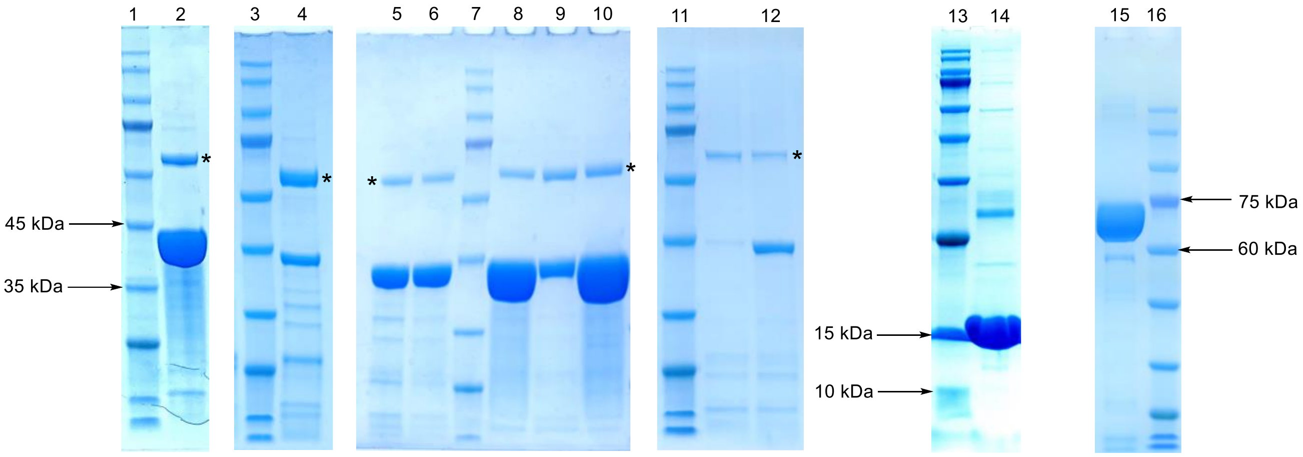

Supplementary Figure 8. Denaturing SDS-PAGE analyses (conc. 10%) of BsfK, BsfK variants, BsfB1 and TF-BsfB2. The asterisks indicate an unknown impure protein associated with the purification of BsfK homologues/variants. Lane 1, Protein standard; Lane 2, BsfK (39.63 kDa); Lane 3, Protein standard; Lane 4, BsfK G163A, Lane 5, BsfK G166A, Lane 6, BsfK S170A; Lane 7, protein standard; Lane 8, BsfK G168A; Lane 9, BsfK D186A; Lane 10, BsfK D187A; Lane 11, protein standard; Lane 12, BsfK K169A; Lane 13, protein standard; Lane 14, BsfB1 (13.68 kDa); Lane 15, TF-BsfB2 (68.18 kDa); Lane 16, Protein standard.

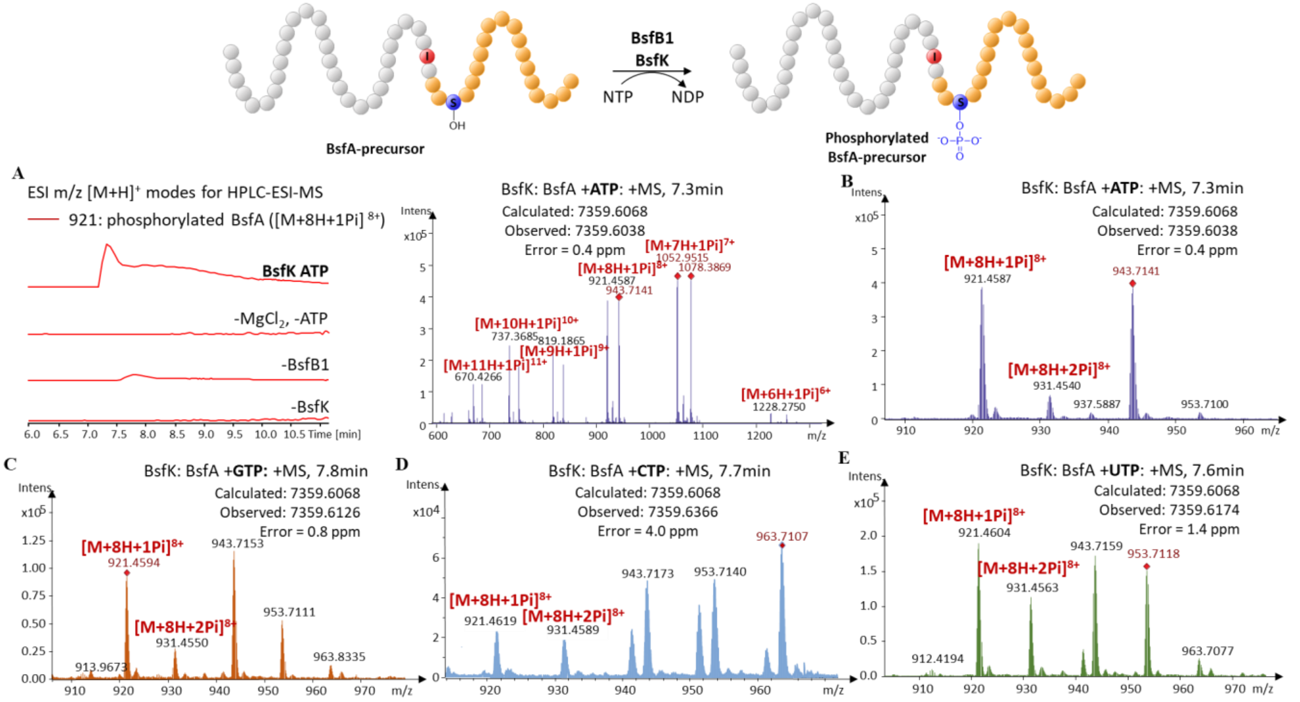

Supplementary Figure 9. *In vitro* characterization of BsfK with ATP and other phosphate donors. (**A**) HPLC-ESI-MS analysis of phosphorylated BsfA generated from biochemical experiments of BsfK (and BsfB1) with ATP as the phosphate donor. (**B**)-(**E**) ESI-HRMS analyses of phosphorylated BsfA with ATP, GTP, CTP and UTP as the phosphate donors, respectively. The masses of 943.71xx is proposed to result from a non-specific gluconic acid derivative attachment.

```
    BsfK   GEGRIDFAELPPSGPKRVEDAGDEYGRFRFSLAMERVFLPLYALFSMPDAVALHG   149
    PcyK   LKPAQHLIEYSIDDCGR........DNLKLGAVVERVALPLYLLLAHRTMLGIHA   135
    VmvK   WRSLRVLWEDSTIYFDG...........PAIEPFVDRVVFPVIESLNPDSVTLHG   117
    LlbK   LLVAHIRYQPAARGSW............SLDTLLERVVLPIALLTTTRPLIALHG   129
    LssK   LPDRHIAYEPPESSDW............ALDTLLERIVLPIALLTTDRPLVALHG   129
    LvtK   LAEGHMGYQAPPHSAW............ALDTLLERIVIPIALLTTDRPLVALHG   128
   PadeK   VQDGSSILVSPFDQAE............EDWVRLFILGTCMGILLLQRKIMPLHG   124
   ThcoK   ITNGNRIEVHAYSGAD............EDRIRLYVLGTCMGALLLQRRILPLHG   120
   BaceK   IQEGKQIIVSPMENTC............EDKIRLYVLGTCMGALLMQRGILPLHG   120
   PapoK   VREGKEIEVSIFSGAD............PDTVRLFVLGTCMGVLLMQRRILPIHG   120
   SyanK   FHGPDLIEIQPYPDTP............VTYLPFPLLGPIMALALHMRGYITLHA   134
    PsmK   IQNGEKIIVSPMKGSD............QDKIRLYILGTCMGALLMQRKILPLHG   120
Consensus                                                       h

    BsfK   SAVVLNG.EAFLFIGRSGAGKSTTAYEFVRRGATLLADDLIVADVARGI.ALGGA   202
    PcyK   STVQIDE.QAWSFVGSSGAGKSTTAGELLKHGGRLVADDLTLYDTSTGS.LLPGA   188
    VmvK   AAIVSKK..AMLLLGQSGVGKSTITRELSGRGWRFWADDVVAINH.....TLLKP   165
    LlbK   SAVHFPQEQAIAIIGDSGAGKSTTALALWRQGASLLADDLVLIDVERQV.ILAGA   183
    LssK   SALRCPGGDTIAIIGDSGAGKSTTALGLVRRGATLLADDLVLIDVERRL.LLAGA   183
    LvtK   SALRCSGNDTIAIIGDSGAGKSTTALGLVRRGATLLADDLVLVDVERRL.LLAGA   182
   PadeK   SAVAIDG.KAYAIIGDSGAGKSTLALHLMSEGYPLLSDDVIPVVMTQGS.PWVVP   177
   ThcoK   SVVARDG.RAYAIVGESGAGKSTMSAALLERGFRLVTDDVAAIVFDERGTPLVMP   174
   BaceK   SAINIDG.KVYAIVGDSGAGKSTLAAAFLSRGYTLLSDDVIAVTVSPGKNPIVIP   174
   PapoK   SAVVIGG.RAYAFVGESGTGKSTLAAAFRQAGYQMVSDDVIAVKATASS.AIVYP   173
   SyanK   SAIDLDG.HGVIFVGDKLAGKSTTAAAFLRAGHRLLTDDLLAIRANPDGALEILP   188
    PsmK   SAIAING.KAYAFVGRSGAGKSTLASALLSKGYQLLSDDVIAVSLSEDNIPFVTP   174
Consensus                 g    gkst        g     dd
```

Supplementary Figure 10. Partial sequence alignment of BsfK with other lasso peptide kinases.

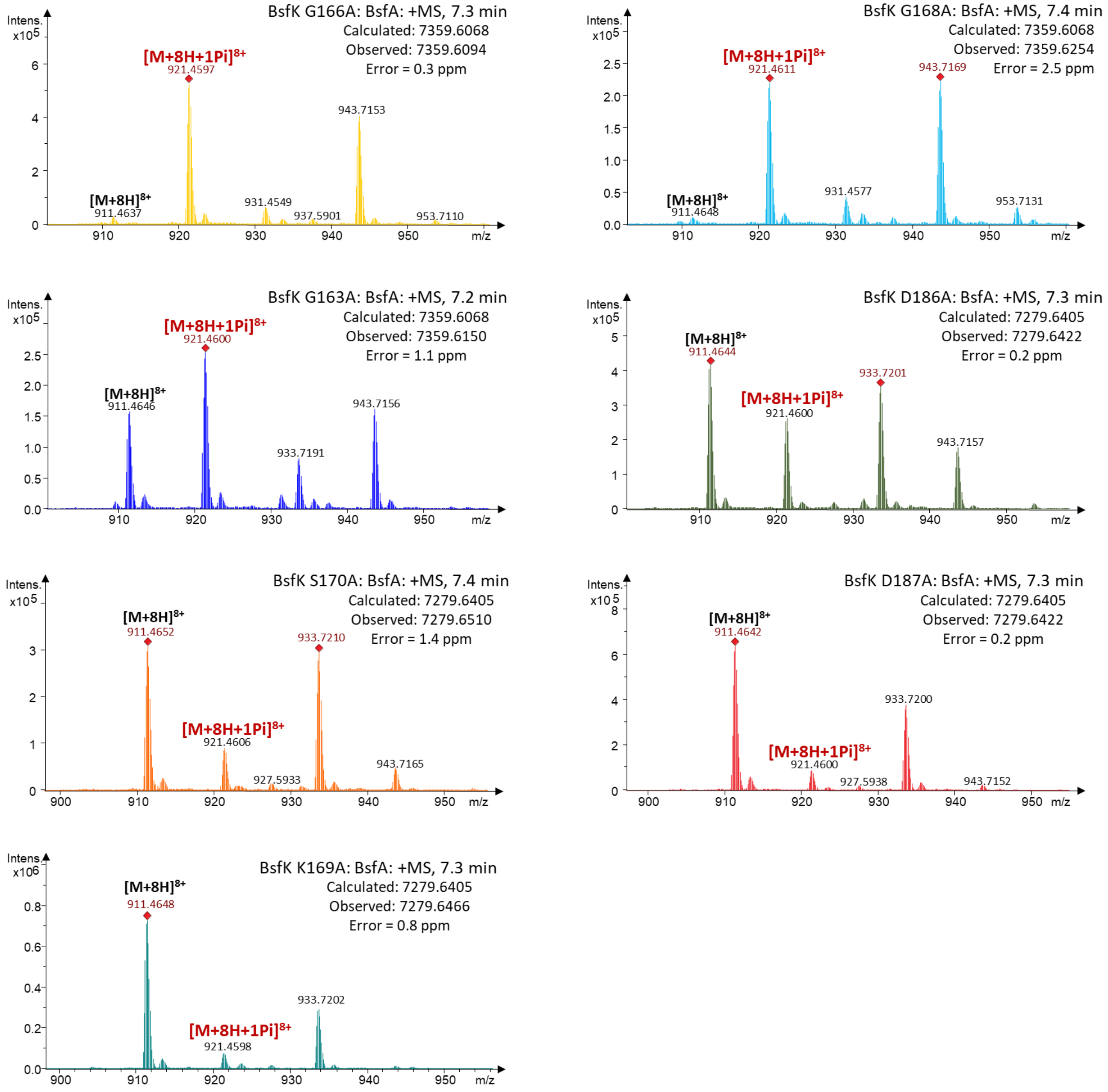

Supplementary Figure 11. *In vitro* characterization of BsfA phosphorylation with different BsfK variants. G166A and G168A have almost the same catalytic activities compared with wild type BsfK, whereas the activities of other variants are diminished in varying degrees.

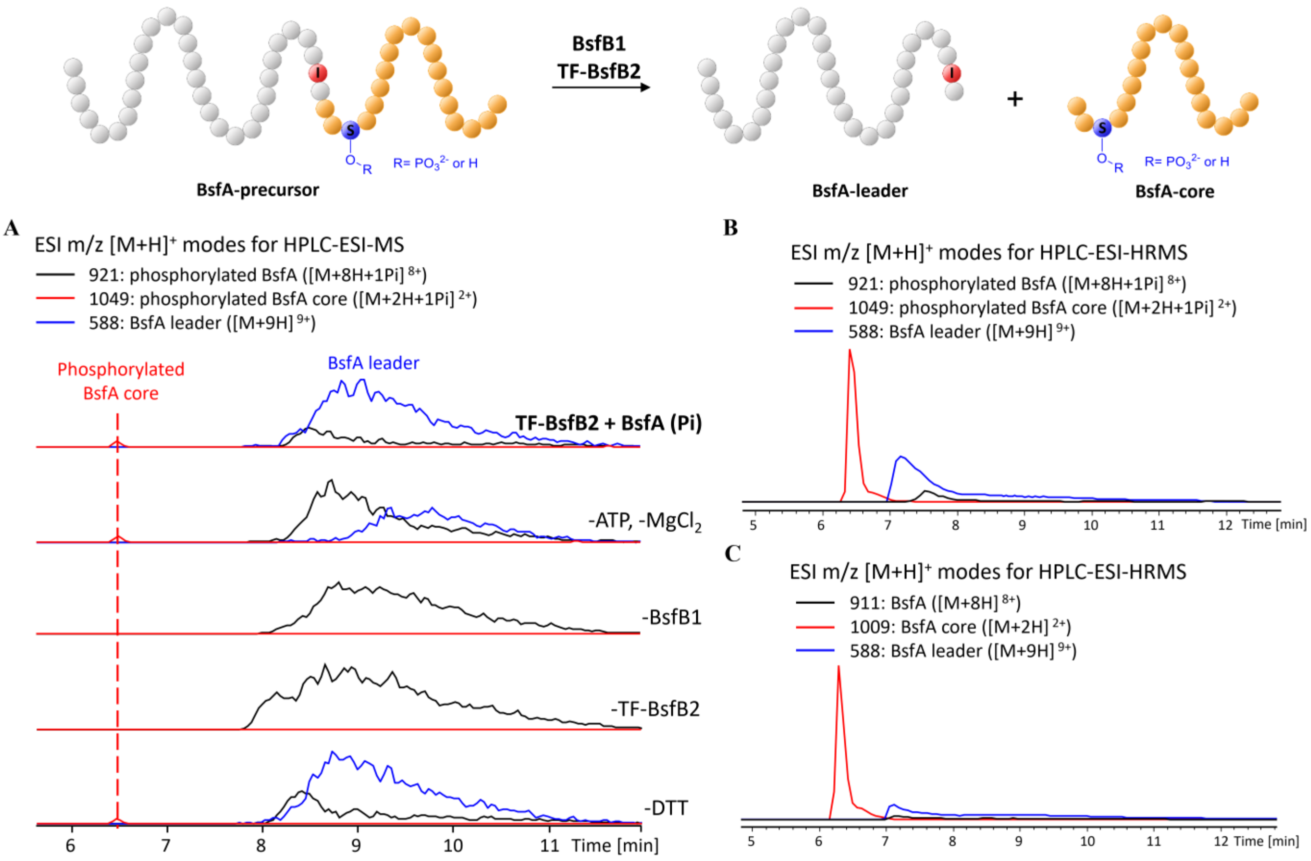

Supplementary Figure 12. *In vitro* characterization of TF-BsfB2 with precursor peptides. (**A**) HPLC-ESI-MS analysis of enzymatic assay for TF-BsfB2 and negative controls. (**B**) HPLC-ESI-HRMS analysis of enzymatic assay for TF-BsfB2 with phosphorylated precursor. (**C**) HPLC-ESI-HRMS analysis of enzymatic assay for TF-BsfB2 with unphosphorylated precursor.

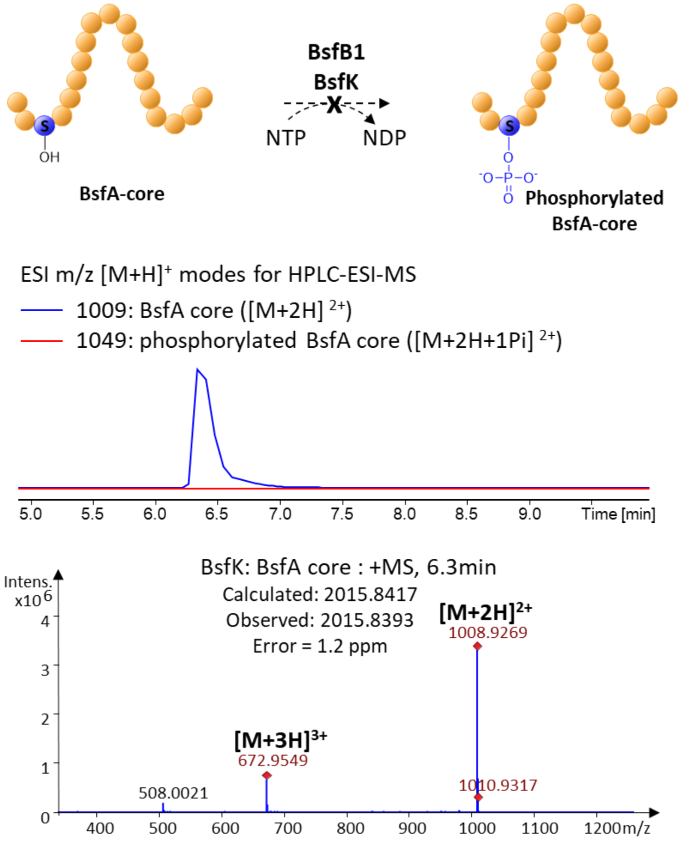

Supplementary Figure 13. *In vitro* characterization of BsfK with BsfA core peptide. Only unphosphorylated core peptide was detected.

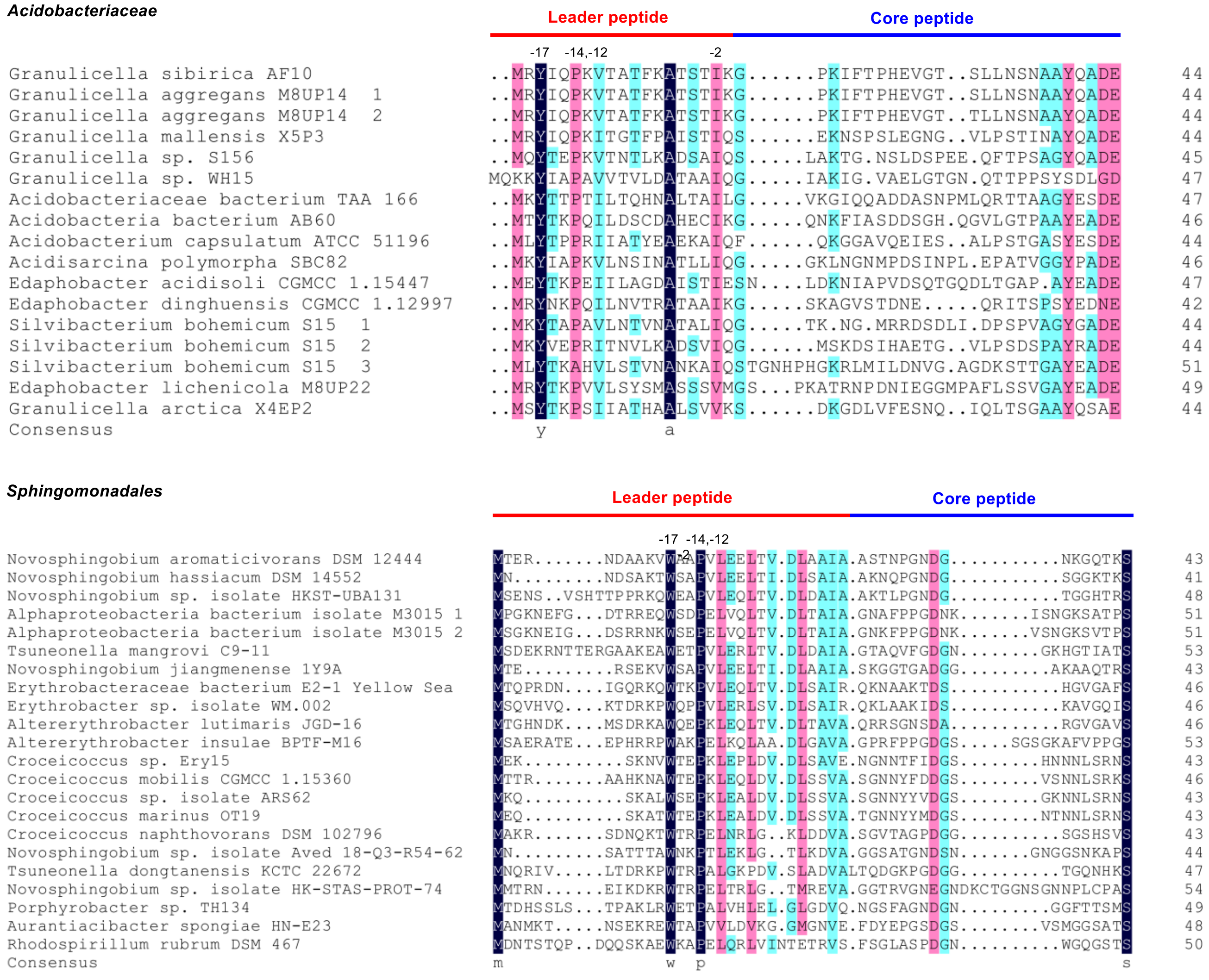

Supplementary Figure 14. Sequence alignments of lasso peptide precursors derived from *Acidobacteriaceae* and *Sphingomonadales*, respectively.

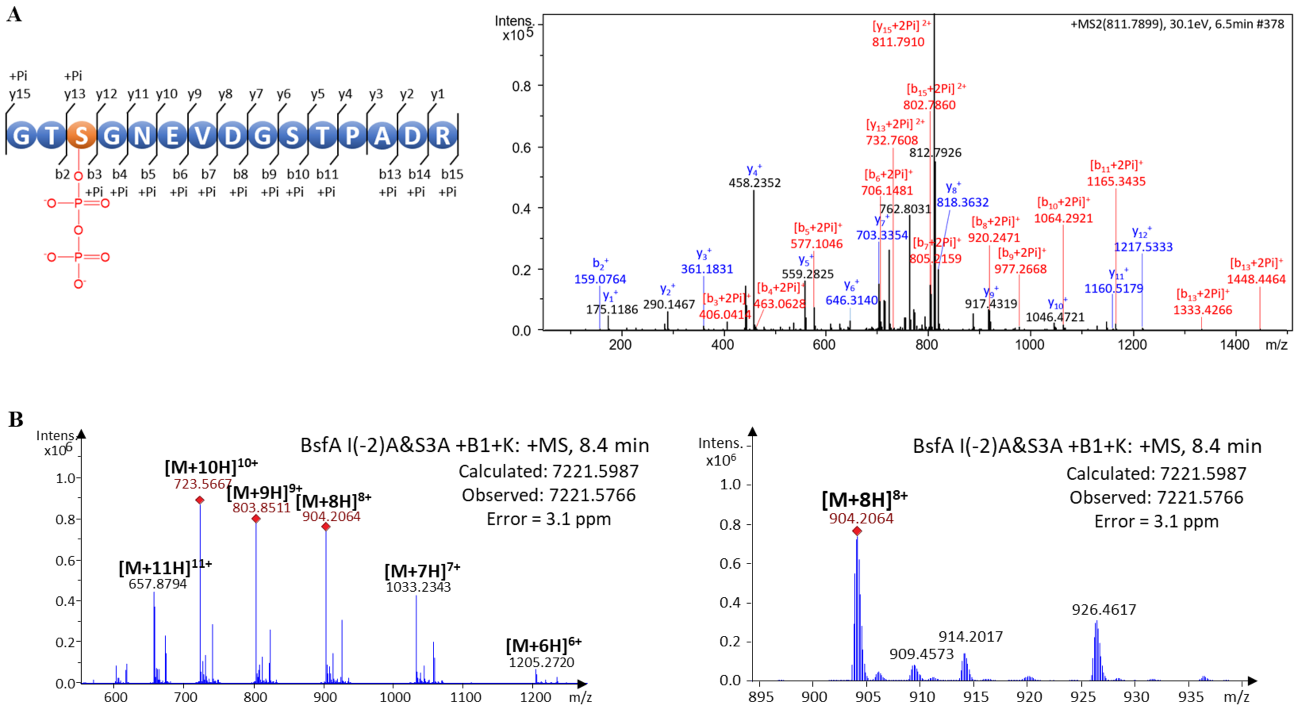

Supplementary Figure 15. Validation of Ser3 as the polyphosphorylated site of BsfA I(-2)A. (**A**) MS/MS fragmentation analysis of the core peptide fragment after trypsin digestion. (**B**) HPLC-ESI-HRMS analysis of BsfA I(-2)A&S3A that co-expressed with BsfB1 and BsfK.

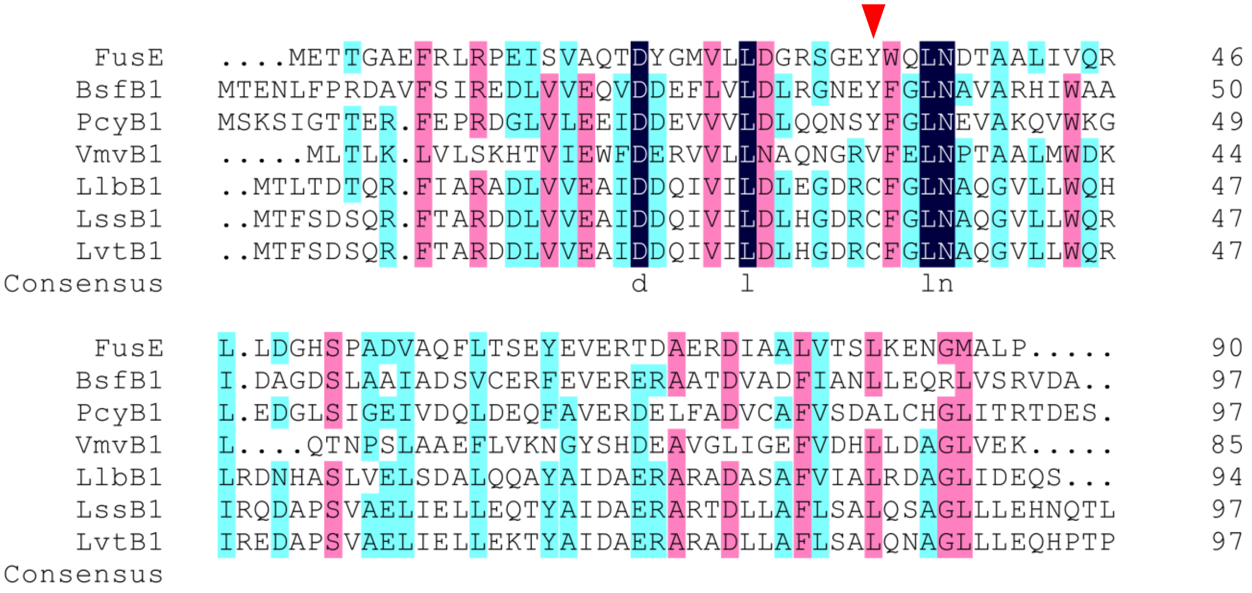

Supplementary Figure 16. Sequence alignment of BsfB1 with FusE and other *Bradymonadales* derived B1 proteins. The Tyr33 residue in FusE is indicated by red triangle.

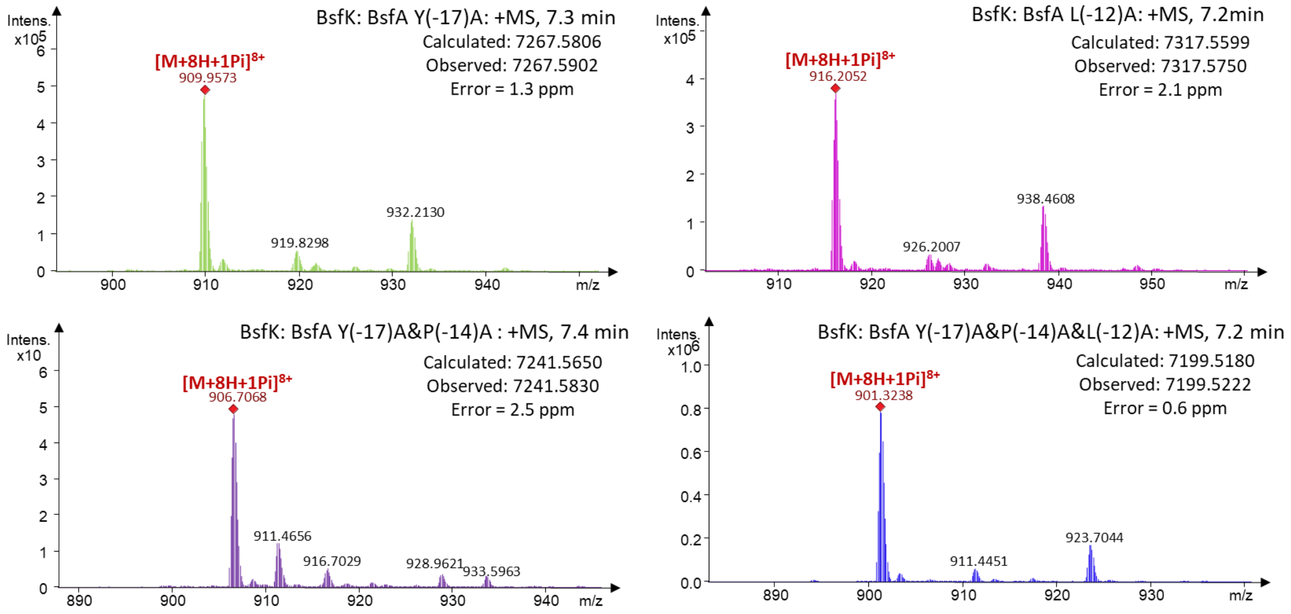

Supplementary Figure 17. *In vitro* characterization of BsfK with BsfA Y(-17)A, L(-12)A, Y(-17)A&P(-14)A and Y(-17)A&P(-14)A&L(-12)A variants, respectively. The P(-14)A variant precipitates immediately after added to the assay of BsfK, and no mass of precursor is observed.

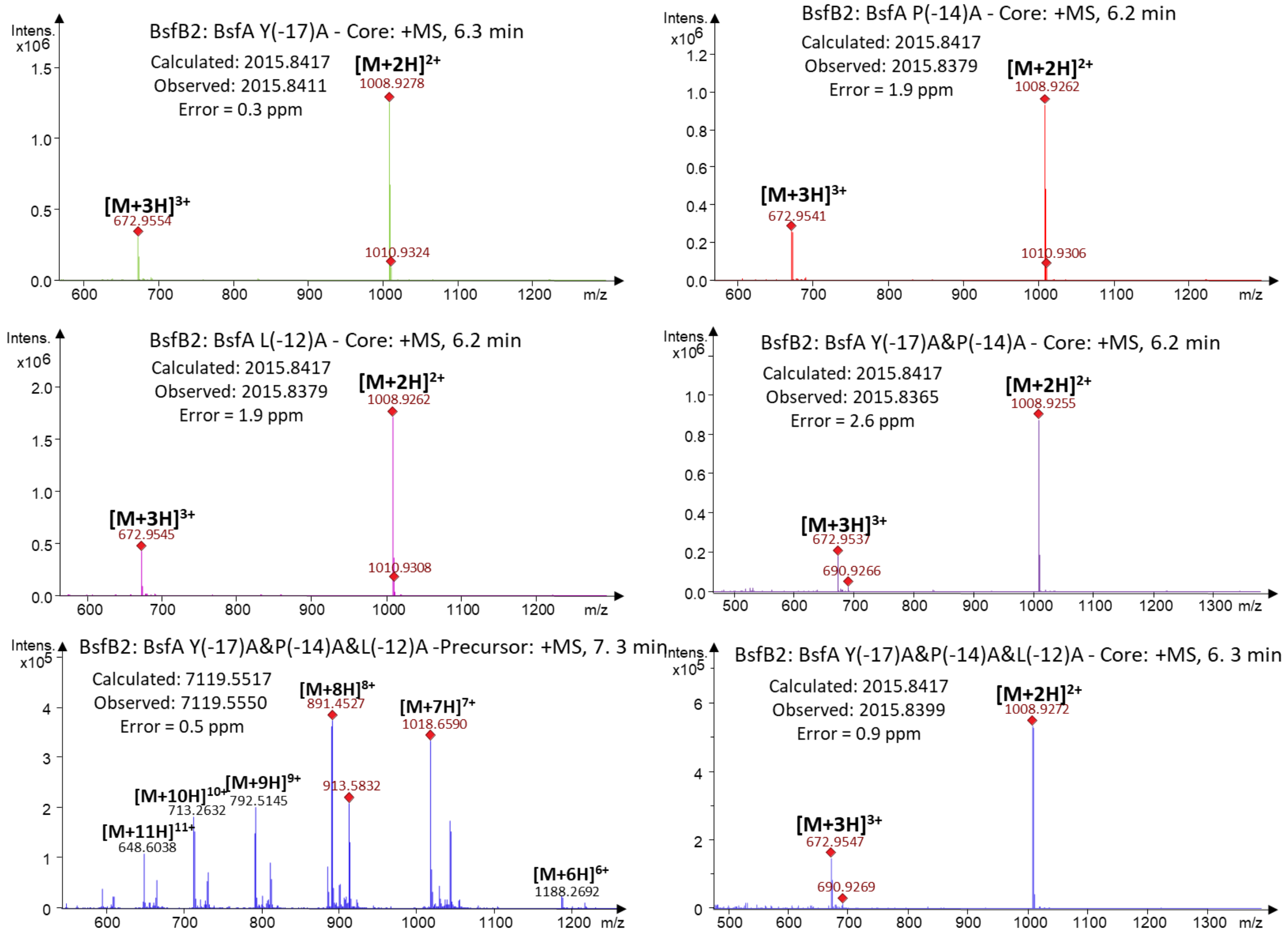

Supplementary Figure 18. *In vitro* characterization of BsfB2 with BsfA Y(-17)A, P(-14)A, L(-12)A, Y(-17)A&P(-14)A and Y(-17)A&P(-14)A&L(-12)A variants, respectively. The mass of core peptide is detected in all the variant assays, whereas the mass of precursor is only detected in the assay of Y(-17)A&P(-14)A&L(-12)A variant.

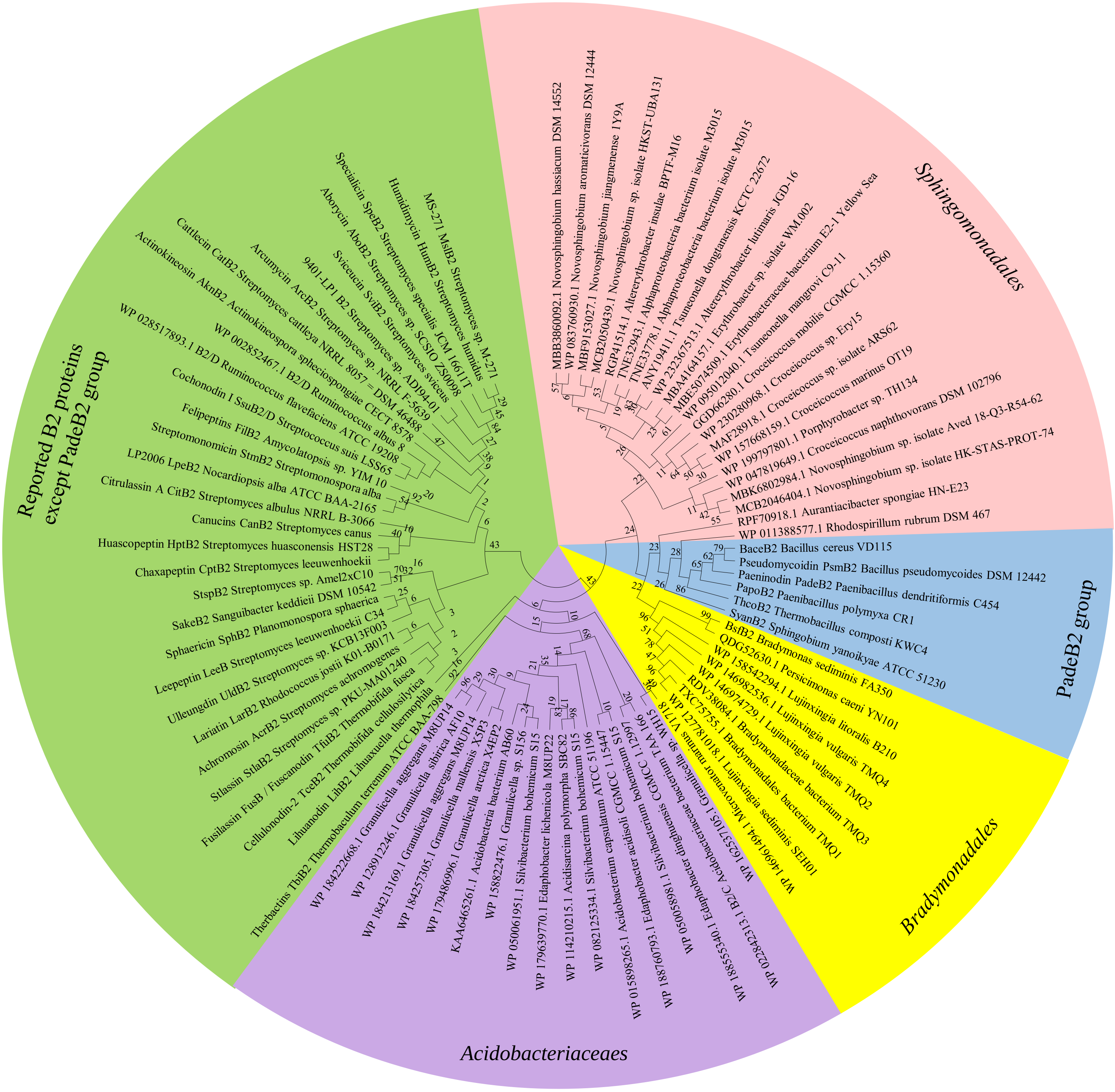

Supplementary Figure 19. Phylogenetic analysis of lasso peptide B2 proteins (please see supporting information 3 for the B2 protein sequences). The tree was constructed using the neighbor-joining method and a bootstrap test with 2000 iterations.

Supplementary Tables

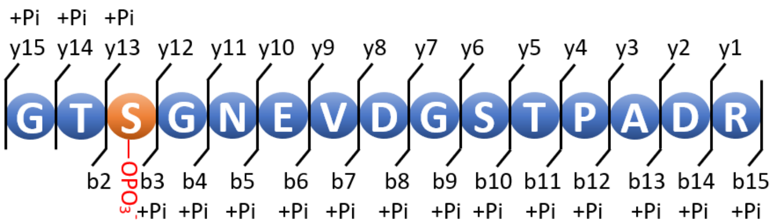

MH+ ion	Calculated mass	Observed mass	Errors (ppm)	MH+ ion	Calculated mass	Observed mass	Errors (ppm)
b1	58.0288	-	-	y15+Pi	1542.6068	771.8082([M+2H]$^{2+}$)	1.5
b2	159.0765	159.0763	1.3	y14+Pi	1485.5853	743.2979([M+2H]$^{2+}$)	2.2
b3+Pi	326.0748	326.0745	0.9	y13+Pi	1384.5377	1384.5207	12.3
b4+Pi	383.0963	383.0962	0.3	y12	1217.5393	1217.5394	0.1
b5+Pi	497.1392	497.1388	0.8	y11	1160.5179	1160.5162	1.5
b6+Pi	626.1818	626.1816	0.3	y10	1046.4749	1046.4756	0.7
b7+Pi	725.2502	725.2502	0.0	y9	917.4323	917.4313	1.1
b8+Pi	840.2771	840.2770	0.1	y8	818.3639	818.3638	0.1
b9+Pi	897.2986	897.2957	3.2	y7	703.3370	703.3368	0.3
b10+Pi	984.3306	984.3311	0.5	y6	646.3155	646.3146	1.4
b11+Pi	1085.3783	1085.3794	1.0	y5	559.2835	559.2830	0.9
b12+Pi	1182.4311	1182.4318	0.6	y4	458.2358	458.2356	0.4
b13+Pi	1253.4682	1253.4650	2.6	y3	361.1831	361.1828	0.8
b14+Pi	1368.4951	1368.4899	3.8	y2	290.1459	290.1460	0.3
b15+Pi	1524.5962	762.8035 ([M+2H]$^{2+}$)	2.3	y1	175.1190	175.1189	0.6

Supplementary Table 1. MS/MS fragmentation analysis of phosphorylated BsfA fragment after trypsin digestion.

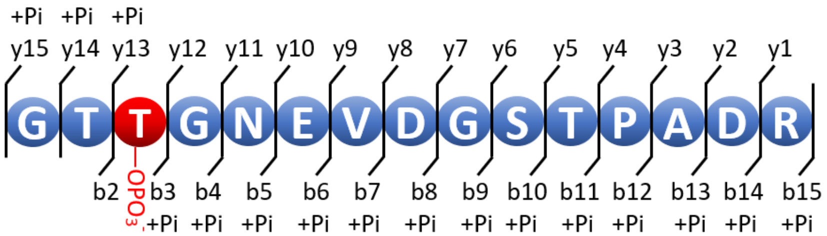

MH+ ion	Calculated mass	Observed mass	Errors (ppm)	MH+ ion	Calculated mass	Observed mass	Errors (ppm)
b1	58.0288	-	-	y15+Pi	1556.6225	778.8170([M+2H]$^{2+}$)	2.7
b2	159.0765	159.0770	3.1	y14+Pi	1499.6010	750.3033([M+2H]$^{2+}$)	1.1
b3+Pi	340.0904	340.0907	0.9	y13+Pi	1398.5533	699.7829([M+2H]$^{2+}$)	3.7
b4+Pi	397.1119	397.1123	1.0	y12	1217.5393	1217.5397	0.3
b5+Pi	511.1548	511.1552	0.8	y11	1160.5179	1160.5214	3.0
b6+Pi	640.1974	640.1985	1.7	y10	1046.4749	1046.4755	0.6
b7+Pi	739.2658	739.2661	0.4	y9	917.4323	917.4329	0.7
b8+Pi	854.2928	854.2928	0.0	y8	818.3639	818.3639	0.0
b9+Pi	911.3143	911.3136	0.8	y7	703.3370	703.3372	0.3
b10+Pi	998.3463	998.3429	3.4	y6	646.3155	646.3161	0.9
b11+Pi	1099.3940	1099.3944	0.4	y5	559.2835	559.2840	0.9
b12+Pi	1196.4467	1196.4400	5.6	y4	458.2358	458.2359	0.2
b13+Pi	1267.4838	1267.4874	2.8	y3	361.1831	361.1840	2.5
b14+Pi	1382.5108	1382.5041	4.8	y2	290.1459	290.1457	0.7
b15+Pi	1538.6119	769.8116([M+2H]$^{2+}$)	2.6	y1	175.1190	175.1191	0.6

Supplementary Table 2. MS/MS fragmentation analysis of phosphorylated BsfA S3T fragment after trypsin digestion.

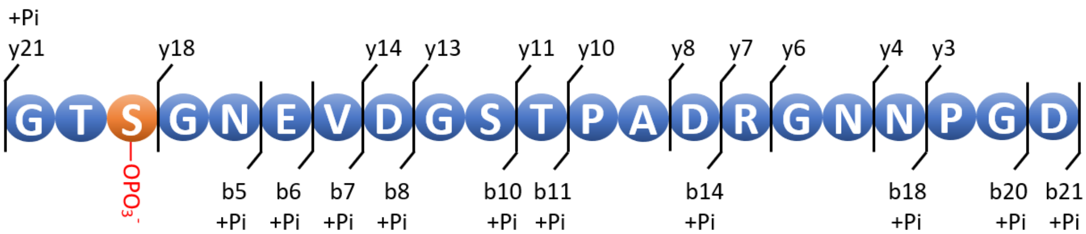

MH+ ion	Calculated mass	Observed mass	Errors (ppm)	MH+ ion	Calculated mass	Observed mass	Errors (ppm)
b1	58.0288	-	-	y21+Pi	2096.8153	1048.9103 ($[M+2H]^{2+}$)	0.9
b2	159.0765	-	-	y20+Pi	2039.7938	-	-
b3+Pi	326.0748	-	-	y19+Pi	1938.7461	-	-
b4+Pi	383.0963	-	-	y18	1771.7478	886.3760 ($[M+2H]^{2+}$)	1.7
b5+Pi	497.1392	497.1362	6.0	y17	1714.7264	-	-
b6+Pi	626.1818	626.1797	3.4	y16	1600.6834	-	-
b7+Pi	725.2502	725.2485	2.3	y15	1471.6408	-	-
b8+Pi	840.2771	840.2739	3.8	y14	1372.5773	1372.5685	6.4
b9+Pi	897.2986	-	-	y13	1257.5473	1257.5373	8.0
b10+Pi	984.3306	984.3245	6.2	y12	1200.5273	-	-
b11+Pi	1085.3783	1085.3744	3.6	y11	1113.4873	1113.4879	0.5
b12+Pi	1182.4311	-	-	y10	1012.4473	1012.4374	9.8
b13+Pi	1253.4682	-	-	y9	915.3873	-	-
b14+Pi	1368.4951	1368.4921	2.2	y8	844.3573	844.3538	4.1
b15+Pi	1524.5962	-	-	y7	729.3273	729.3227	6.3
b16+Pi	1581.6177	-	-	y6	573.2273	573.2252	3.7
b17+Pi	1695.6606	-	-	y5	516.2073	-	-
b18+Pi	1809.7036	905.3555($[M+2H]^{2+}$)	0.1	y4	402.1573	402.1597	6.0
b19+Pi	1906.7563	-	-	y3	288.1173	288.1182	3.1
b20+Pi	1963.7778	982.3901($[M+2H]^{2+}$)	2.5	y2	191.0673	-	-
b21+Pi	2078.8047	1039.9089($[M+2H]^{2+}$)	2.8	y1	134.0473	-	-

Supplementary Table 3. MS/MS fragmentation analysis of phosphorylated BsfA core peptide generated by TF-BsfB2.

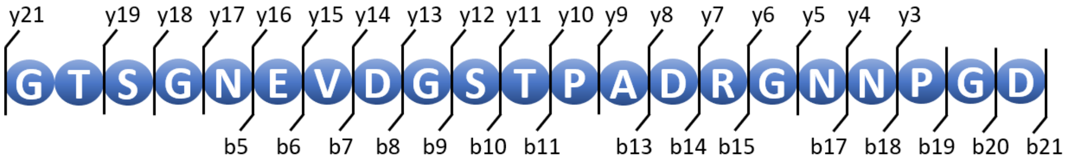

MH+ ion	Calculated mass	Observed mass	Errors (ppm)	MH+ ion	Calculated mass	Observed mass	Errors (ppm)
b1	58.0288	-	-	y21	2016.8490	1008.9264([M+2H]$^{2+}$)	1.7
b2	159.0765	-	-	y20	1959.8275	-	-
b3	246.1085	-	-	y19	1858.7798	929.8926([M+2H]$^{2+}$)	1.0
b4	303.1300	-	-	y18	1771.7478	886.3692([M+2H]$^{2+}$)	9.4
b5	417.1729	417.1726	0.7	y17	1714.7264	857.8650([M+2H]$^{2+}$)	2.1
b6	546.2155	546.2140	2.7	y16	1600.6834	800.8403([M+2H]$^{2+}$)	6.3
b7	645.2839	645.2818	3.3	y15	1471.6408	1471.6365	2.9
b8	760.3108	760.3076	4.2	y14	1372.5773	1372.5769	0.3
b9	817.3323	817.3325	0.2	y13	1257.5473	1257.5395	6.2
b10	904.3643	904.3594	5.4	y12	1200.5273	1200.5130	11.9
b11	1005.4120	1005.4088	3.2	y11	1113.4873	1113.4874	0.1
b12	1102.4648	-	-	y10	1012.4473	1012.4398	7.4
b13	1173.5019	1173.4944	6.4	y9	915.3873	915.3897	2.6
b14	1288.5288	1288.5251	2.9	y8	844.3573	844.3515	6.9
b15	1444.6299	1444.6140	11.0	y7	729.3273	729.3234	5.3
b16	1501.6514	-	-	y6	573.2273	573.2237	6.3
b17	1615.6943	808.3524([M+2H]$^{2+}$)	2.0	y5	516.2073	516.1991	15.9
b18	1729.7373	865.3684([M+2H]$^{2+}$)	4.5	y4	402.1573	402.1603	7.5
b19	1826.7900	913.9008([M+2H]$^{2+}$)	2.4	y3	288.1173	288.1176	1.0
b20	1883.8115	942.4097([M+2H]$^{2+}$)	0.3	y2	191.0673	-	-
b21	1998.8384	999.9220([M+2H]$^{2+}$)	0.8	y1	134.0473	-	-

Supplementary Table 4. MS/MS fragmentation analysis of BsfA core peptide generated by TF-BsfB2.

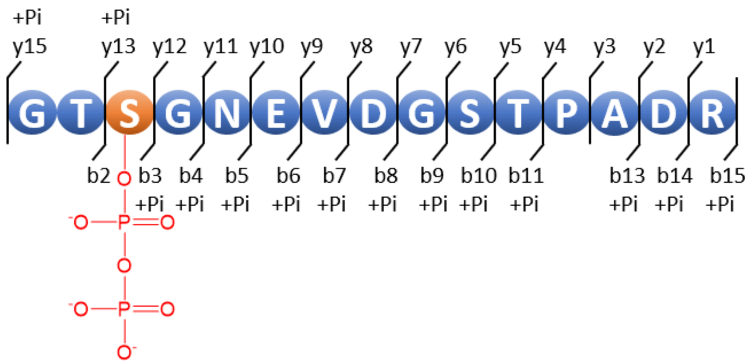

MH+ ion	Calculated mass	Observed mass	Errors (ppm)	MH+ ion	Calculated mass	Observed mass	Errors (ppm)
b1	58.0288	-	-	y15+2Pi	1622.5731	811.7910([M+2H]$^{2+}$)	1.0
b2	159.0765	159.0764	0.6	y14+2Pi	1565.5516	-	-
b3+2Pi	406.0411	406.0427	3.9	y13+2Pi	1464.5040	732.7608([M+2H]$^{2+}$)	7.0
b4+2Pi	463.0626	463.0628	0.4	y12	1217.5393	1217.5333	4.9
b5+2Pi	577.1055	577.1046	1.6	y11	1160.5179	1160.5179	0.0
b6+2Pi	706.1481	706.1481	0.0	y10	1046.4749	1046.4721	2.7
b7+2Pi	805.2165	805.2159	0.7	y9	917.4323	917.4319	0.4
b8+2Pi	920.2434	920.2471	4.0	y8	818.3639	818.3632	0.9
b9+2Pi	977.2649	977.2668	1.9	y7	703.3370	703.3354	2.3
b10+2Pi	1064.2969	1064.2921	4.5	y6	646.3155	646.3140	2.3
b11+2Pi	1165.3446	1165.3435	0.9	y5	559.2835	559.2825	1.8
b12+2Pi	1262.3974	-	-	y4	458.2358	458.2352	1.3
b13+2Pi	1333.4345	1333.4266	5.9	y3	361.1831	361.1834	0.8
b14+2Pi	1448.4614	1448.4464	10.4	y2	290.1459	290.1467	2.8
b15+2Pi	1604.5625	802.7845 ([M+2H]$^{2+}$)	0.5	y1	175.1190	175.1186	2.3

Supplementary Table 5. MS/MS fragmentation analysis of polyphosphorylated BsfA I(-2)A fragment after trypsin digestion.

Primer	Sequence (5'-3')
BsfK-for	TATCATATGGAAAACACCGACCGTCT
BsfK-rev	TATCTCGAGTTATTTAGAAGCCAGACCAA
BsfB1-for	TATCCATGGGCCGTGAAACCCCGATGACC
BsfB1-rev	TATAAGCTTTTAAGCGTCAACACGAGAAA
TF-BsfB2-for	TATCATATGAAACCGATCCACCCGAT
TF-BsfB2-rev	TATCTCGAGTTAAGCAGCAACTTCACGGA
BsfA T2A-for	GCTATCATCCGTGGTGCATCTGGTAACGAAGTT
BsfA T2A-rev	AACTTCGTTACCAGATGCACCACGGATGATAGC
BsfA S3A-for	ATCATCCGTGGTACCGCAGGTAACGAAGTTGAC
BsfA S3A-rev	GTCAACTTCGTTACCTGCGGTACCACGGATGAT
BsfA S10A-for	AACGAAGTTGACGGTGCAACCCCGGCTGACCGT
BsfA S10A-rev	ACGGTCAGCCGGGGTTGCACCGTCAACTTCGTT
BsfA T11A-for	GAAGTTGACGGTTCTGCACCGGCTGACCGTGGT
BsfA T11A-rev	ACCACGGTCAGCCGGTGCAGAACCGTCAACTTC
BsfA S3T-for	ATCATCCGTGGTACCACCGGTAACGAAGTTGAC
BsfA S3T-rev	GTCAACTTCGTTACCGGTGGTACCACGGATGAT
BsfA S3Y-for	ATCATCCGTGGTACCTATGGTAACGAAGTTGAC
BsfA S3Y-rev	GTCAACTTCGTTACCATAGGTACCACGGATGAT
BsfA I(-2)T-for	ACCCTGGCTGCTATCACCCGTGGTACCTCTGGT
BsfA I(-2)T-rev	ACCAGAGGTACCACGGGTGATAGCAGCCAGGGT
BsfA I(-2)A-for	ACCCTGGCTGCTATCGCACGTGGTACCTCTGGT
BsfA I(-2)A-rev	ACCAGAGGTACCACGTGCGATAGCAGCCAGGGT
BsfA I(-2)A&S3A-for	ACCCTGGCTGCTATCGCACGTGGTACCGCAGGTAACGAAGTTGAC
BsfA I(-2)A&S3A-rev	GTCAACTTCGTTACCTGCGGTACCACGTGCGATAGCAGCCAGGGT
BsfA I(-2)L-for	ACCCTGGCTGCTATCCTGCGTGGTACCTCTGGT
BsfA I(-2)L-rev	ACCAGAGGTACCACGCAGGATAGCAGCCAGGGT
BsfA I(-2)V-for	ACCCTGGCTGCTATCGTTCGTGGTACCTCTGGT
BsfA I(-2)V-rev	ACCAGAGGTACCACGAACGATAGCAGCCAGGGT
BsfA Y(-17)A-for	TCTCGTAAACTGATCGCAAAAAAACCGCAGCTG
BsfA Y(-17)A-rev	CAGCTGCGGTTTTTTTGCGATCAGTTTACGAGA
BsfA P(-14)A-for	CTGATCTACAAAAAAGCACAGCTGACCTACCAC
BsfA P(-14)A-rev	GTGGTAGGTCAGCTGTGCTTTTTTGTAGATCAG
BsfA L(-12)A-for	TACAAAAAACCGCAGGCAACCTACCACTCTACC
BsfA L(-12)A-rev	GGTAGAGTGGTAGGTTGCCTGCGGTTTTTTGTA
BsfA Y(-17)A&P(-14)A-for	TCTCGTAAACTGATCGCAAAAAAAGCACAGCTGACCTACCAC
BsfA Y(-17)A&P(-14)A-rev	GTGGTAGGTCAGCTGTGCTTTTTTTGCGATCAGTTTACGAGA
BsfA Y(-17)A&P(-14)A&L(-12)A-for	TCTCGTAAACTGATCGCAAAAAAAGCACAGGCAACCTACCACTCTACC
BsfA Y(-17)A&P(-14)A&L(-12)A -rev	GGTAGAGTGGTAGGTTGCCTGTGCTTTTTTTGCGATCAGTTTACGAGA

BsfK G163A-for	GCTTTCCTGTTCATCGCACGTTCTGGTGCTGGT
BsfK G163A-rev	ACCAGCACCAGAACGTGCGATGAACAGGAAAGC
BsfK G166A-for	TTCATCGGTCGTTCTGCAGCTGGTAAATCTACC
BsfK G166A-rev	GGTAGATTTACCAGCTGCAGAACGACCGATGAA
BsfK G168A-for	GGTCGTTCTGGTGCTGCAAAATCTACCACCGCT
BsfK G168A-rev	AGCGGTGGTAGATTTTGCAGCACCAGAACGACC
BsfK S170A-for	TCTGGTGCTGGTAAAGCAACCACCGCTTACGAA
BsfK S170A-rev	TTCGTAAGCGGTGGTTGCTTTACCAGCACCAGA
BsfK D186A-for	GCTACCCTGCTGGCTGCAGACCTGATCGTTGCT
BsfK D186A-rev	AGCAACGATCAGGTCTGCAGCCAGCAGGGTAGC
BsfK D187A-for	ACCCTGCTGGCTGACGCACTGATCGTTGCTGAC
BsfK D187A-rev	GTCAGCAACGATCAGTGCGTCAGCCAGCAGGGT
BsfK H148A-for	GACGCTGTTGCTCTGGCAGGTTCTGCTGTTGTT
BsfK H148A-rev	AACAACAGCAGAACCTGCCAGAGCAACAGCGTC
BsfK K169A-for	CGTTCTGGTGCTGGTGCATCTACCACCGCTTAC
BsfK K169A-rev	GTAAGCGGTGGTAGATGCACCAGCACCAGAACG

Supplementary Table 6. Primers used in this study.
